# BDNF and TRiC-inspired reagent rescue cortical synaptic deficits in a mouse model of Huntington’s disease

**DOI:** 10.1016/j.nbd.2024.106502

**Published:** 2024-04-10

**Authors:** Yingli Gu, Alexander Pope, Charlene Smith, Christopher Carmona, Aaron Johnstone, Linda Shi, Xuqiao Chen, Sarai Santos, Claire Cecile Bacon-Brenes, Thomas Shoff, Korbin M. Kleczko, Judith Frydman, Leslie M. Thompson, William C. Mobley, Chengbiao Wu

**Affiliations:** a Department of Neurology, The Fourth Hospital of Harbin Medical University, 150001, China; b Department of Neurosciences, University of California San Diego, La Jolla, CA 92093, United States of America; c Department of Psychiatry and Human Behavior, University of California, Irvine, CA 92697, United States of America; d Department of Bioengineering, University of California San Diego, La Jolla, CA 92093, United States of America; e Beckman Laser Institute & Medical Clinic, University of California, Irvine, Irvine, CA, United States; f Department of Biomedical Engineering, University of California, Irvine, Irvine, CA, United States; g Neuroscience, Scripps College, Claremont, CA 91711, United States of America; h Department of Biology and Genetics, Stanford University, Stanford, CA 94305-5430, United States of America; i Institute of Memory Impairments and Neurological Disorders, University of California, Irvine, CA 92697, United States of America; j Department of Neurobiology and Behavior, University of California, Irvine, CA 92697, United States of America; k Sue and Bill Gross Stem Cell Center, University of California, Irvine, CA 92697, United States of America

**Keywords:** Huntington’s disease, BACHD, Brain-derived neurotrophic factor, Synapse, Neuronal activity, Multi-array electrode, PSD95, Pre- and post-synaptic, Cortical neurons, Long term culture, TRiC

## Abstract

Synaptic changes are early manifestations of neuronal dysfunction in Huntington’s disease (HD). However, the mechanisms by which mutant HTT protein impacts synaptogenesis and function are not well understood. Herein we explored HD pathogenesis in the BACHD mouse model by examining synaptogenesis and function in long term primary cortical cultures. At DIV14 (days in vitro), BACHD cortical neurons showed no difference from WT neurons in synaptogenesis as revealed by colocalization of a pre-synaptic (Synapsin I) and a post-synaptic (PSD95) marker. From DIV21 to DIV35, BACHD neurons showed progressively reduced colocalization of Synapsin I and PSD95 relative to WT neurons. The deficits were effectively rescued by treatment of BACHD neurons with BDNF. The recombinant apical domain of CCT1 (ApiCCT1) yielded a partial rescuing effect. BACHD neurons also showed culture age-related significant functional deficits as revealed by multielectrode arrays (MEAs). These deficits were prevented by BDNF, whereas ApiCCT1 showed a less potent effect. These findings are evidence that deficits in BACHD synapse and function can be replicated in vitro and that BDNF or a TRiC-inspired reagent can potentially be protective against these changes in BACHD neurons. Our findings support the use of cellular models to further explicate HD pathogenesis and potential treatments.

## 1. Introduction

Huntington’s disease (HD) is a fatal disorder impacting movement and cognition due to expanded CAG repeats (>36) within the huntingtin gene. The mutant protein (mHTT) thus contains an expanded polyglutamine tract (Group, 1993; Mangiarini et al., 1996; Sharp et al., 1995). HD is marked by severe cortical-striatal degeneration with clinical manifestations including chorea and other changes in movement, cognitive decline and neuropsychiatric disturbances(Barron et al., 2021; Finkbeiner, 2011; McColgan and Tabrizi, 2018; Roos, 2010; Vonsattel and DiFiglia, 1998; Walker, 2007). Only pharmacological interventions exist for alleviating symptoms(Caron et al., 2018; Novak and Tabrizi, 2011; Roos, 2010) with no disease modifying treatments currently available (Caron et al., 2018; Finkbeiner, 2011; Frank, 2014; Komatsu, 2021; Lane et al., 2018; Pan and Feigin, 2021).

Accumulation of mHTT disrupts protein homeostasis (Caron et al., 2018; Cepeda and Levine, 2022; Frank, 2014; Lane et al., 2018). mHTT is known to impact many facets of neuronal function including gene expression, mitochondria, endoplasmic reticulum (ER), protein turnover, axonal transport, and synaptic transmission (Bennett et al., 2007; Browne, 2008; Buren et al., 2016; Cepeda and Levine, 2022; Guo et al., 2016; Leitman et al., 2013; Li et al., 2003; Mackay et al., 2023; Maity et al., 2022; Santos et al., 2010; Schmidt et al., 2018; Virlogeux et al., 2018; Wu et al., 2023; Xie et al., 2010; Yoshii and Constantine-Paton, 2010).

Synaptic homeostasis is critical for neuronal structure and function. Synaptic dysfunction marks neurodegenerative disorders, including HD (Barry et al., 2022; Y. Chen et al., 2019; Cummings et al., 2007; Kolodziejczyk et al., 2014; Lepeta et al., 2016; Milnerwood et al., 2006; Milnerwood and Raymond, 2007; Murphy et al., 2000; Nithianantharajah and Hannan, 2013; Schirinzi et al., 2016; Selkoe, 2002; Subramanian and Tremblay, 2021; Taoufik et al., 2018; Veldman and Yang, 2018), as it contributes directly to neuronal circuit dysfunction leading to cognitive, behavioral and motor deficits(Y. Chen et al., 2019; Cummings et al., 2007; Dallerac et al., 2011; Kavalali et al., 1999; Kolodziejczyk et al., 2014; Kovalenko et al., 2018; Lepeta et al., 2016; Li et al., 2003; Morton et al., 2001; Nithianantharajah and Hannan, 2013; Selkoe, 2002; Smith-Dijak et al., 2019; Subramanian and Tremblay, 2021).

A prominent clinical manifestation of HD is the structural and functional degeneration of cortical-striatal pathway. Striatal medium spiny neuron (MSNs) degeneration is conjoined with loss of cortical pyramidal neurons (Vonsattel and DiFiglia, 1998). Increasing evidence links cortical-striatal atrophy and degeneration to deficiency in brain-derived neurotrophic factor (BDNF) signaling (Baydyuk and Xu, 2014; Gauthier et al., 2004; Hong et al., 2016; Su et al., 2014; Yu et al., 2018). BDNF is synthesized and released in many brain regions, except striatum (Conner et al., 1997; Hofer et al., 1990; Lindsay et al., 1994; Maisonpierre et al., 1990; Yan et al., 1997). Striatal neurons is dependent on BDNF for critical trophic support (Baydyuk and Xu, 2014; Ferrer et al., 2000; Johnstone and Mobley, 2020; Lu et al., 2014; Moya-Alvarado et al., 2023; Yoshii and Constantine-Paton, 2010; Yu et al., 2018; Zhao et al., 2016; Zuccato and Cattaneo, 2007, 2009). Reduced BDNF in HD is viewed as contributing to dysfunction and degeneration of MSNs and thus the cortical-striatal pathway (Blumenstock and Dudanova, 2020; Cummings et al., 2007; Dallerac et al., 2011; Kovalenko et al., 2018; Milnerwood et al., 2006; Milnerwood and Raymond, 2007; Murphy et al., 2000; Nithianantharajah and Hannan, 2013; Puigdellivol et al., 2015). We have previously demonstrated that BACHD cortical neurons showed deficits in anterograde transport of BDNF that may contribute to atrophy of striatal neurons (Zhao et al., 2016). Our study thus argued for a presynaptic deficit in BDNF transport and release as responsible for striatal atrophy in HD (Zhao et al., 2016). Deciphering the pathogenesis of HD will benefit from studies to explore the roles that BDNF plays in synaptogenesis and synapse maintenance.

Our previous study also established that failed proteostasis is likely linked to reduced BDNF transport and release in HD (Zhao et al., 2016). TRiC is a cytosolic chaperonin (Frydman, 2001; Frydman et al., 1992; Kalisman et al., 2012; Leitner et al., 2012) that contributes to protein homeostasis and quality control (Gestaut et al., 2019; Roh et al., 2015; Thulasiraman et al., 1999). mHTT binds to the apical domain of subunit CCT1 (ApiCCT1) (Kabir et al., 2011; Nollen et al., 2004; Shen et al., 2016; S. Tam et al., 2006; Stephen Tam et al., 2009), and increased expression of CCT prevents mHTT aggregation and toxicity (Kitamura et al., 2006; Rockabrand et al., 2007; Sontag et al., 2013; S. Tam et al., 2006; Stephen Tam et al., 2009). Conversely, reduction of CCT levels enhances mHTT aggregation(Kitamura et al., 2006; Rockabrand et al., 2007; S. Tam et al., 2006; Stephen Tam et al., 2009). Exogenous application of ApiCCT1 reduces aggregation of mHTT (Sontag et al., 2013) and remodels mHTT aggregates(Sahl et al., 2016). Further, ApiCCT1 restored BDNF axonal trafficking in cortical neurons (Zhao et al., 2016). These studies suggest failed proteostasis as one mechanism could contribute to HD pathogenesis via reduction in BDNF secretion (Zhao et al., 2016). However, it is not clear whether or not TRiC reagents such as ApiCCT1 also have a role in synaptic function in HD.

Since cortical dysfunction is also a significant clinical manifestation of HD pathology, we asked if HD cortical neurons showed impairment in synaptogenesis and neuronal function in vitro. We used long-term in vitro cultures of cortical neurons of WT and the BACHD HD mouse model (Gray et al., 2008) to track synaptogenesis and neuronal activity. We demonstrated that, although BACHD cortical neurons formed functional synapses, they developed significant deficits in synapse maintenance and neuronal activities in an age-dependent manner. Furthermore, we showed that BDNF, to a lesser extent ApiCCT1, rescued these deficits in BACHD neurons.

### 1.1. Ethical statement

All experiments involving the use of laboratory animals have been approved by the Institutional Animal Care and Use Committee of University of California San Diego (Protocol# S15159). Surgical and animal procedures were carried out strictly following the NIH Guide for the Care and Use of Laboratory Animals.

## 2. Materials and methods

### 2.1. Reagents and antibodies

10× Hanks’ Balanced Salt solution (HBSS), 2.5% Trypsin-EDTA (10×), 100× Penicillin-Streptomycin, 100× Glutamax, 50× B27, Neuronal basal media were all purchased from Invitrogen. DNase I grade II from bovine pancreas (Roche, Cat#10104159001) was dissolved in 1× HBSS at 10 mg/ml (10×) and filter sterilized. FBS was from Mediatech Inc. (Cat# 35-010-CV). 0.1% Poly-l-Lysine (Cultrex^®^ Poly-l-Lysine) was from Trevigen (Gaithersburg, MD; Cat# 3438-100-01). Human recombinant BDNF was from Genentech (San Francisco, CA). Hoechst 33258 was from Sigma (Cat#861405). All other chemicals and lab wares were from Bio-Rad, Fisher, Sigma, VWR. Purified recombinant ApiCCT1 was provided by Dr. J. Frydman of Stanford University.

Mouse anti-PSD95 (Clone 28/47 from Biolegend, Cat. #810401), rabbit monoclonal anti-Synapsin I (Cell Signaling, Cat. #D12G5), mouse anti-synaptobrevin 2 (104211) (SYSY; 1:5000 dilution), mouse anti-synaptophysin (PA1-1043; 1:5000 dilution), mouse anti-synaptotagmin (610,434; 1:2000 dilution) (BD), mouse anti-SNAP25 (ADI-VAM-SV012; 1:2000 dilution) (Enzo), mouse anti-β-actin was from Santa Cruz Biotechnology (Cat. #47778, 1:2000 dilution). Secondary goat anti-rabbit, goat anti-mouse antibodies conjugated to Alexa-488, 568 were from Invitrogen. All antibodies were used at dilutions as instructed by the suppliers. Paraformaldehyde (PFA) was purchased from Sigma.

### 2.2. Neuronal culture and maintenance

Established protocols were followed to set up cortical neurons collected from mouse E18 embryos(Fang et al., 2017; Zhao et al., 2016). Briefly, cortical tissues were extracted from E18 pregnant mice and BACHD and WT embryos (Gray et al., 2008; Zhao et al., 2016). Tissues were rinsed in HBSS with 1% Penicillin-Streptomycin, followed by dissociation in 0.25% trypsin with 1 mg/ml DNase I. Cortical neurons were isolated and plated with plating media (Neurobasal with 10% FBS, 1xB27,1xGlutaMAX) onto either glass coverslips for immunostaining or into 12 well plates for biochemistry. The plating medium was replaced with maintenance medium (Neurobasal, 1xB27, 1xGlutamax) 24 h after. Only 2/3 of the media was replaced every other day prior to experiments.

### 2.3. Immunostaining and confocal microscopy

E18 cortical neurons from wild type (WT), BACHD mice were cultured on coverglasses that were precoated with 0.1% poly-d-Lysine (1 h at RT). Cultures from the same batch of dissection at DIV7 (Days-In-Vitro), DIV14, DIV21, DIV28 and DIV35 were rinsed and were fixed in 4% PFA for 15 mins at RT. The samples were washed 3 times with PBS and were blocked and permeabilized in 5% goat serum containing 0.2% TritonX-100 for 15 mins at RT. Neurons were first incubated with a mouse anti-PSD95 antibody (1:200 dilution in PBS) for 3 h at RT. The samples were washed 3 times (5 min each) in PBS and were incubated with the rabbit monoclonal antibody against Synapsin I (1:200 dilution in PBS) at 4 °C overnight. Samples were then washed in PBS 3 times (5 min each) and were incubated with goat anti-mouse Alexa 488 and goat anti-rabbit Alexa 568 secondary antibody conjugates (both at 1:600 dilution in PBS) for 1 h at RT. Hoechst 33258 (1.0 μg/ml in PBS) was used to stain nuclei for 5 min at RT. Samples were washed and mounted onto slides. Images of neurons (20–30 per condition) were captured with a Leica SP confocal microscope under a 63× oil objective lens with a 1.6× zoom factor.

We followed published methods (Paul et al., 2013; Rizk et al., 2014) to quantify synaptic formation in cortical neurons of WT and BACHD. Segmentation was done on the neuronal images to identify puncta of PSD95 and Synapsin I by using Squassh in the NIH MOSAIC image suite (Paul et al., 2013; Rizk et al., 2014). The raw confocal images consisted of both somas/processes and were evaluated using ImageJ/MOSAIC Suite. For each channel, the background values were subtracted, and the images were then segmented using the Squassh function to isolate puncta. We used the Pearson correlation coefficient (PCC) to measure co-localization of PSD95/Synapsin I to define synaptogenesis. PCC measures the strength of a linear association between two variables: the distribution of PSD95 vs Synapsin I signals. The r values for PCC ranges from +1 to −1. A value <0 indicates a negative association. A value of 0 indicates no association between PSD95 and Synapsin I signals. An r value >0 indicates a positive association between PSD95 and Synapsin I signals. An r value of 1 indicates a complete overlap between PSD95 and Synapsin I signals. ImageJ was also used to measure the soma size and the size of PSD95, Synapsin I puncta. Synaptic density normalized against the length of neurites per 100 μm was also analyzed.

### 2.4. BDNF treatment

To test the effect of synaptic deficits by BDNF, exogenous BDNF was included in the neuronal maintenance media. WT or BACHD cortical neuronal cultures were treated with media containing either BDNF (50 ng/ml, final concentration) or vehicle starting at either DIV7 or DIV14 and maintained until DIV21. Neurons were thus treated for either 14 days or 7 days respectively. Synaptic staining (PSD95/Synapsin I) and quantitation of PCCs were carried out at DIV21 as described above.

### 2.5. ApiCCT1 treatment

Recombinant ApiCCT1 was purified as previously described (Shen et al., 2016). Prior to addition to the culture media, the ApiCCT1 protein preparations were desalted and reconstituted into Neurobasal media with the use of Zeba^™^ Spin Desalting Columns, 7 K MWCO, 0.5 mL (ThermoFisher, Cat# 89882). *Re*-purified ApiCCT1 was added to the culture media at a final concentration of 0.1 μM at DIV14. The media were changed with fresh ApiCCT1 every other day prior to analysis at DIV21.

### 2.6. BDNF ELISA

Rapid ELISA Kits for measuring mature BDNF (Cat# BEK-2211-2P) were purchased from Biosensis Pty Ltd. (51 West Thebarton Road, Thebarton, South Australia). Conditioned media were collected from the same batch WT and BACHD cortical neuronal cultures at DIV14 then again at DIV21. The media (~400 μl) were lyophilized overnight, reconstituted with 120 μl sterile dH_2_O by vigorous vortex at RT. Samples were centrifuged at 14 k rpm at 4 °C for 10 min. 100 μl clear supernatants were removed and the amounts of BDNF were measured and quantified against the standard curve following the manufacture’s instruction. To normalize BDNF *sec*retion on a total protein base, we collected neurons from each well, solubilized in radio-immunoprecipitation assay (RIPA) buffer and centrifuged to produce the supernatants. The protein concentrations were determined by BCA method using a NanoDrop^™^ 2000/2000c Spectrophotometer (ThermoFisher) and the results were used to normalize the levels of BDNF in the secreted media.

### 2.7. Multielectrode array (MEA)

WT and BACHD neurons were seeded on PDL-precoated CytoView MEA 24 well plates (Axion Biosystems) at a density of 100,000 cells/well in 10 μl of plating medium. Once neurons were settled, 500 μl of plating medium was added to each well. The exact protocols, as described earlier, were followed to maintain the culture.

At DIV14, the first recording on the Maestro Edge was performed for a total 10 min using the AxIS Navigator Software v3.2.3.1. After recording, the medium was changed to include BDNF (50 ng/ml), ApiCCT1 (0.1 μM) or Vehicle control. The plates were returned to the incubator. At DIV28, A second and final recording was performed. Analysis was performed by running Batch process in AxIS Navigator v2.0.4.21, “re-recording” data for consistency at the end of each experiment. For spike detection, the adaptive threshold crossing was used. The burst detection setting had a maximum inter spike interval of 100 ms and minimum of 5spikes/burst required. For network statistics, the minimum number of spikes required was 30 with at least 35% of the well electrodes participating in the network activity.

Specifically, the following 12 metrics of neuronal activities were captured: 1) weighted mean firing rate (wMFR: average spikes per sec per active electrode) to estimate the overall population excitability and connectivity; 2) Inter-spike intervals (ISIs) coefficient of variation (ISI CoV) to measure irregularity of spike trains; 3) synchrony index to measure the degree to which activity is synchronized; 4) total number of bursts; 5) burst frequency; 6) numbers of spikes/burst; 7) inter-burst intervals (IBIs) to measure the length of quiescent periods between bursts; 8) percentage of network bursts – i.e. the number of spikes in network bursts divided by the total number of spikes, multiplied by 100; 9) numbers of spikes/network burst; 10) the mean ISI within a burst to detect changes in burst patterns; 11) network bursts, to measure periodic and synchronized activity of cultured neurons; and 12) network inter-burst intervals (IBIs) coefficient of variation (IBI CoV).

### 2.8. Western blot analysis and quantification

Parallel sets of primary cortical neurons from the same batch BACHD and WT littermates were cultured and collected at DIV21 and at DIV28. After lysis in RIPA buffer containing PMSF protease inhibitor on ice for 30 min, cell lysates were centrifuged at 13,300 rpm at 4 °C for 10 min. The protein extracts were denatured in SDS sample buffer at 95 °C for 4 min and loaded onto 10–12.5% SDS-PAGE for separation. Separated proteins were electro-transferred onto cellulose nitrate membranes, blocked in 5% skim milk diluted in TBST at RT for 1 h. The antibodies used include: mouse primary antibodies against synaptophysin (Synaptic Systems GmbH, 1:1000), synaptobrevin (mouse, 1:5000), synaptotagmin (mouse, 1:2000), SNAP25 (mouse, 1:5000), PSD95 (mouse, 1:1000) and β-actin (C4) (mouse, sc-47,778 from Sant Cruz) at 4 °C overnight, and then incubated in corresponding secondary antibodies (goat anti-mouse, 1:5000 or 1:10,000). The blots were washed and developed in ECL-Clarity (BioRad). The blots were imaged using ChemiDoc XRS+ (Bio-Rad) and quantitated using ImageLab 6.0.1 software (BioRad). Protein levels were expressed as the ratio of each immunoreactive band and the levels of β-actin.

### 2.9. Statistical analysis

Significance analysis was carried out using Prism. Significances were calculated using either unpaired *t*-test (Mann-Whitney), One Way ANOVA (Dunnett’s post-test) or Two Way ANONA (Tukey’s multiple comparison). These methods are specifically indicated in relevant Figures and Tables. Values are the mean ± SEM of at least three different and independent experiments. All data and *p* values are indicated in the corresponding figures and tables. ns: non significance; * *p* < 0.05; **p < 0.01; *** *p* < 0.001; **** *p* < 0.0001.

## 3. Results

### 3.1. BACHD cortical neurons show culture age-related deficits in synapse maintenance

To determine if BACHD neurons showed deficits in synaptogenesis and synapse formation over time, we cultured E18 cortical neurons, a time point that cortical tissues can be cleanly separated from other brain regions. We maintained the neuronal cultures for up to 35 days in vitro (DIV35) to compare BACHD with WT neurons and track age-dependent changes.

**Fig. 1.**
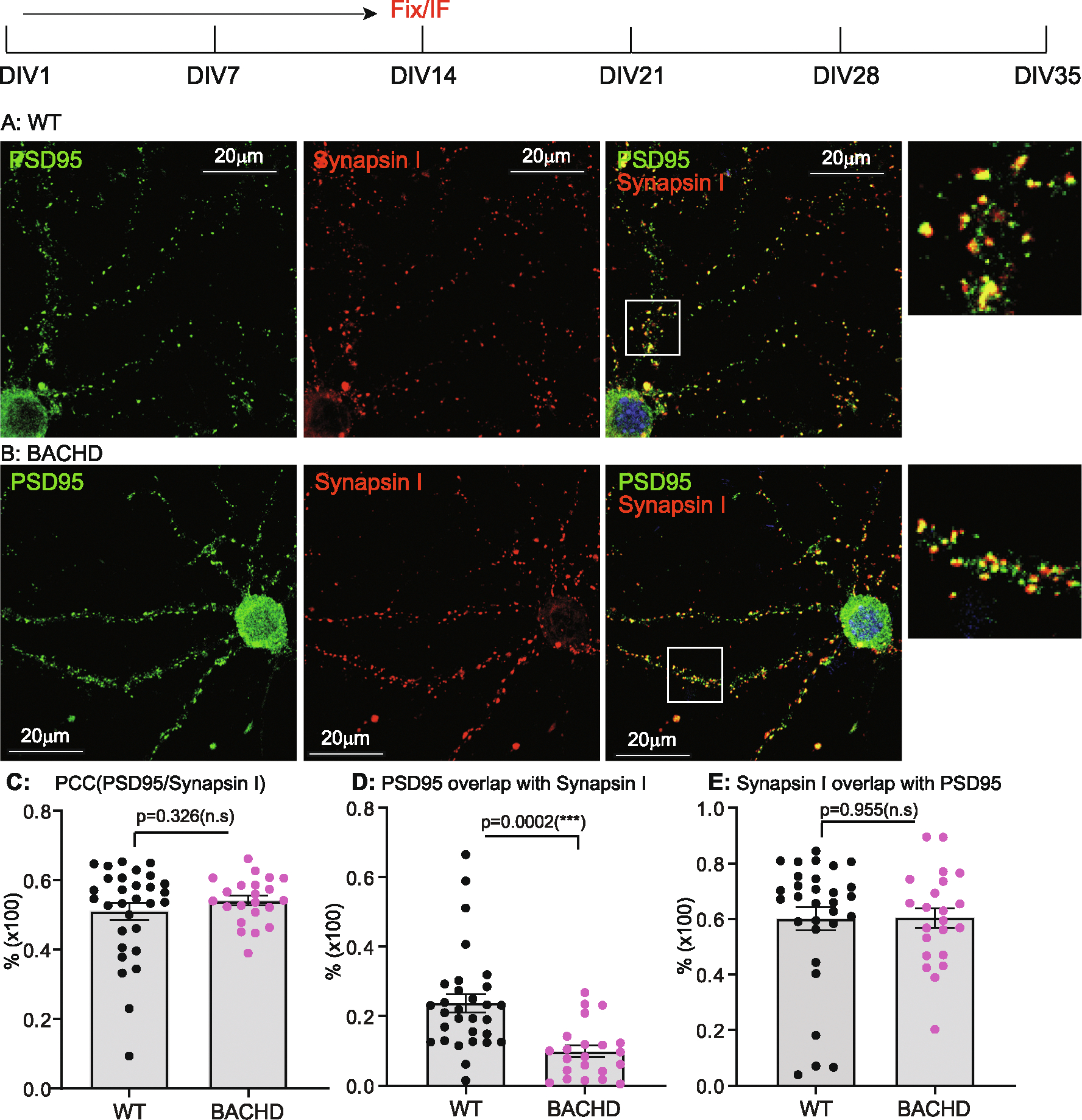
Synaptic analysis of cortical neurons from WT, BACHD at DIV14. E18 cortical neurons from WT, BACHD were dissected, cultured on PLL-coated cover-glasses and maintained as described in Materials and Methods. At DIV14, a set of samples from each genotype were fixed and immunostained. Nuclei were stained with Hoechst 33258. The images were captured under a 63× oil objective using a Leica SP6 confocal microscope. Representative images are shown and co-localization between Synapsin I and PSD95 was quantitated using ImageJ Suite Plugin. Representative images WT (**A**), BACHD (**B**) cortical neurons stained for PSD95 (green) and Synapsin I (red). Regions of interest marked by white boxes are magnified and shown on the right. **C**: Comparison of post- and presynaptic marker colocalization using PCC. **D**: Analysis of the % of PSD95/Synapsin I. **E**: Analysis of the % of Synapsin I/PSD95. Results are shown as mean ± SEM. The numbers of images were analyzed: *n* = 30 for WT, *n* = 22 for BACHD. The data represents at least 4–5 independent cultures. Significance analysis was carried out using Prism. Statistical significances were calculated by Sidak’s multiple comparisons test of One-Way ANOVA. n.s. = non significance. All *p* values are shown.

**Fig. 2.**
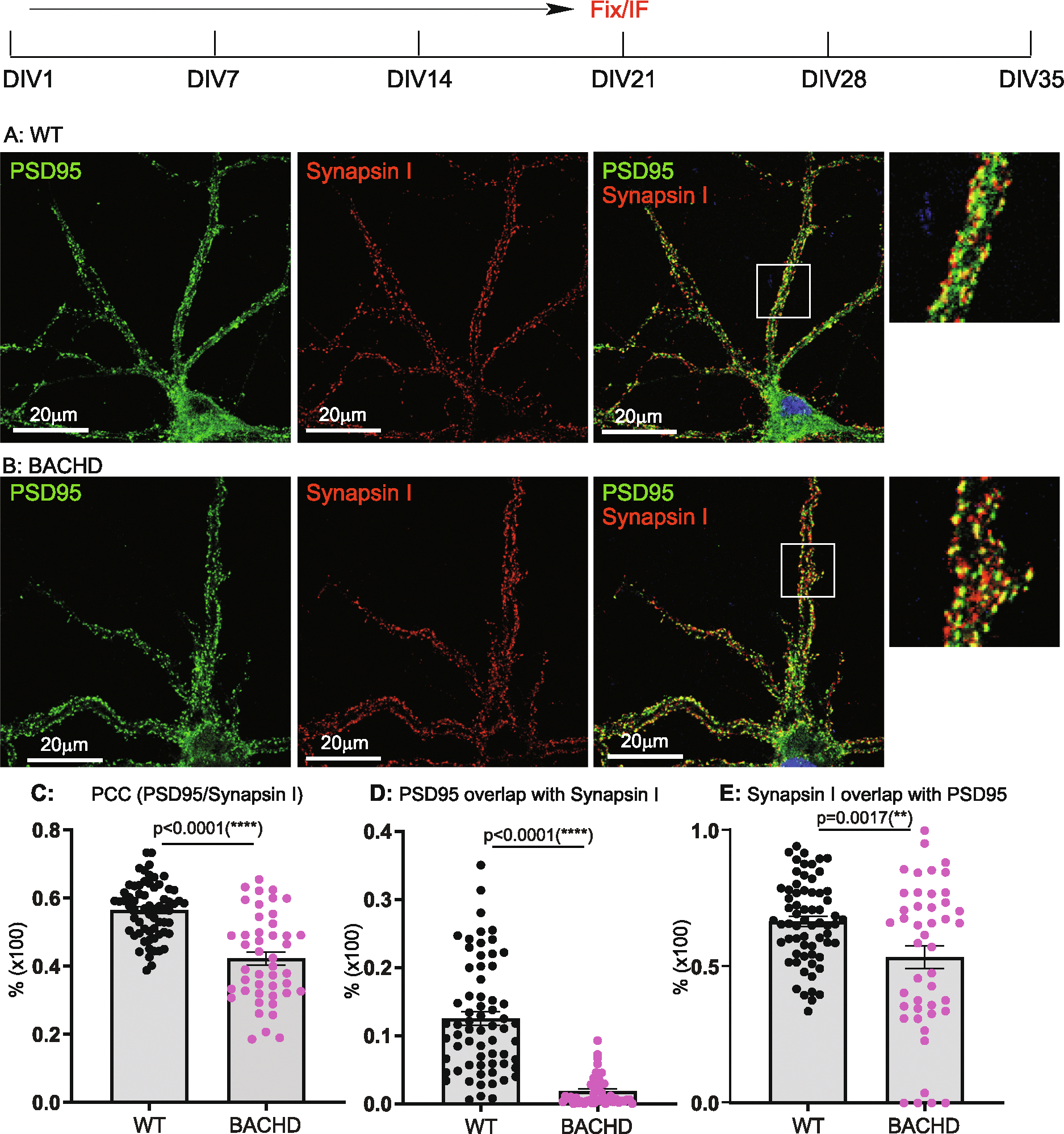
Synaptic analysis of cortical neurons from WT, BACHD at DIV21. Neuronal culture, immunostaining and quantitation are as described in Fig. 1. Representative images of PSD95 (green) and Synapsin I (red) staining in WT (**A**), BACHD (**B**) cortical neurons. Regions of interest marked by white boxes are magnified and shown on the right. **C**: Comparison of post- and presynaptic marker colocalization using PCC. **D**: Analysis of the % of PSD95/Synapsin I. **E**: Analysis of the % of Synapsin I/PSD95. Results are shown as mean ± SEM. The numbers of images were analyzed: *n* = 65 for WT, *n* = 45 for BACHD. The data represents at least 4–5 independent cultures. Significance analysis was carried out using Prism. Statistical significances were calculated by Sidak’s multiple comparisons test of One-Way ANOVA. n.s. = non significance. All *p*-values are shown.

**Fig. 3.**
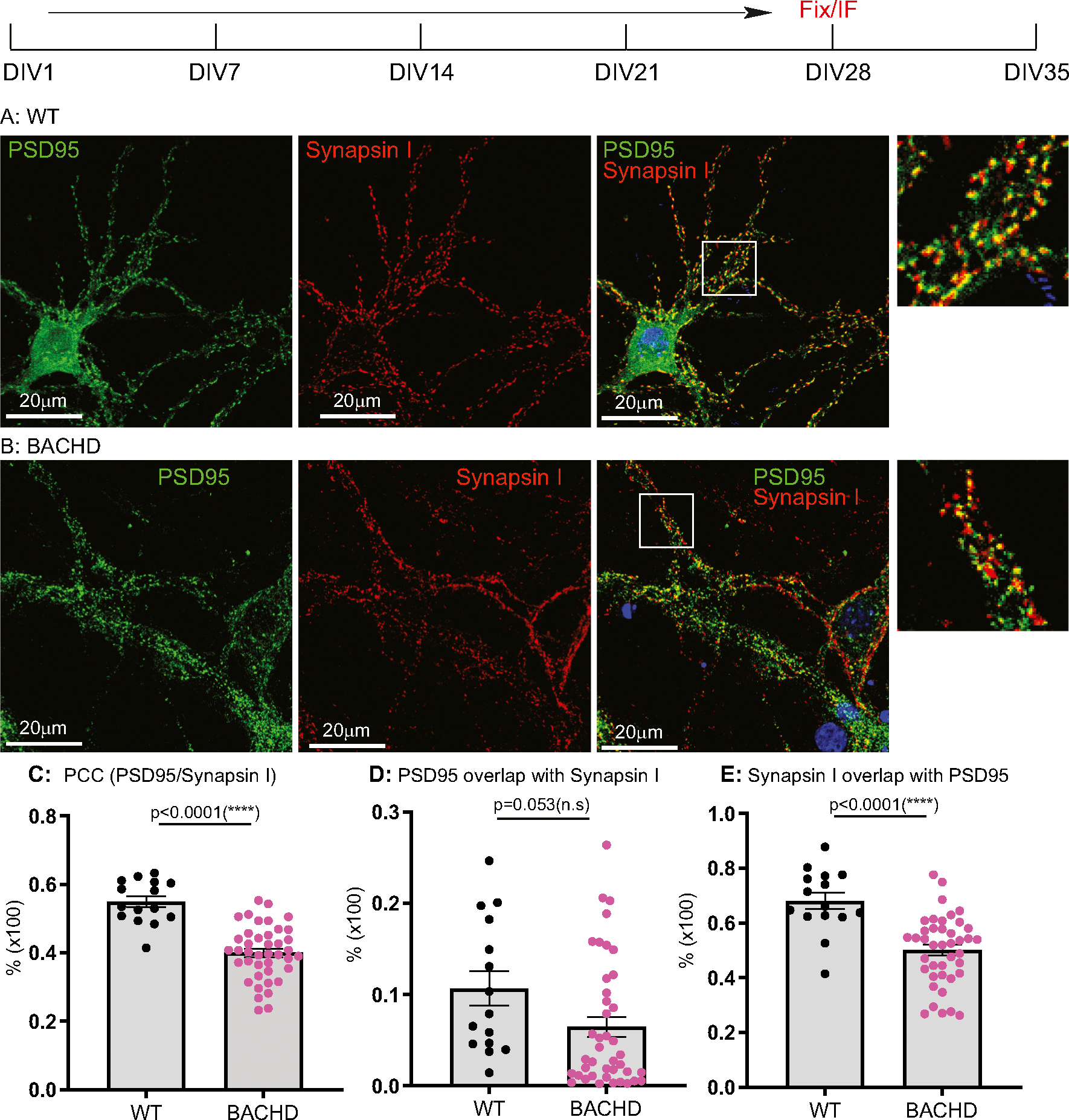
Synaptic analysis of cortical neurons from WT, BACHD at DIV28. Neuronal culture, immunostaining and quantitation are described as in Fig. 1. Representative images of PSD95 (green) and Synapsin I (red) staining respectively in WT (**A**), BACHD (**B**) cortical neurons. White boxes demarcating regions of interest magnified on the right. **C**: Comparison of post- and presynaptic marker colocalization using PCC. **D**: Analysis of the % of PSD95/Synapsin I. **E**: Analysis of the % of Synapsin I/PSD95. Results are shown as mean ± SEM. The numbers of images were analyzed for *n* = 15 (WT), *n* = 41 for BACHD. The data represents at least 4–5 independent cultures. Significance analysis was carried out using Prism. Statistical significances were calculated by Sidak’s multiple comparisons test of One-Way ANOVA. n.s. = non significance. All *p* values are shown.

**Fig. 4.**
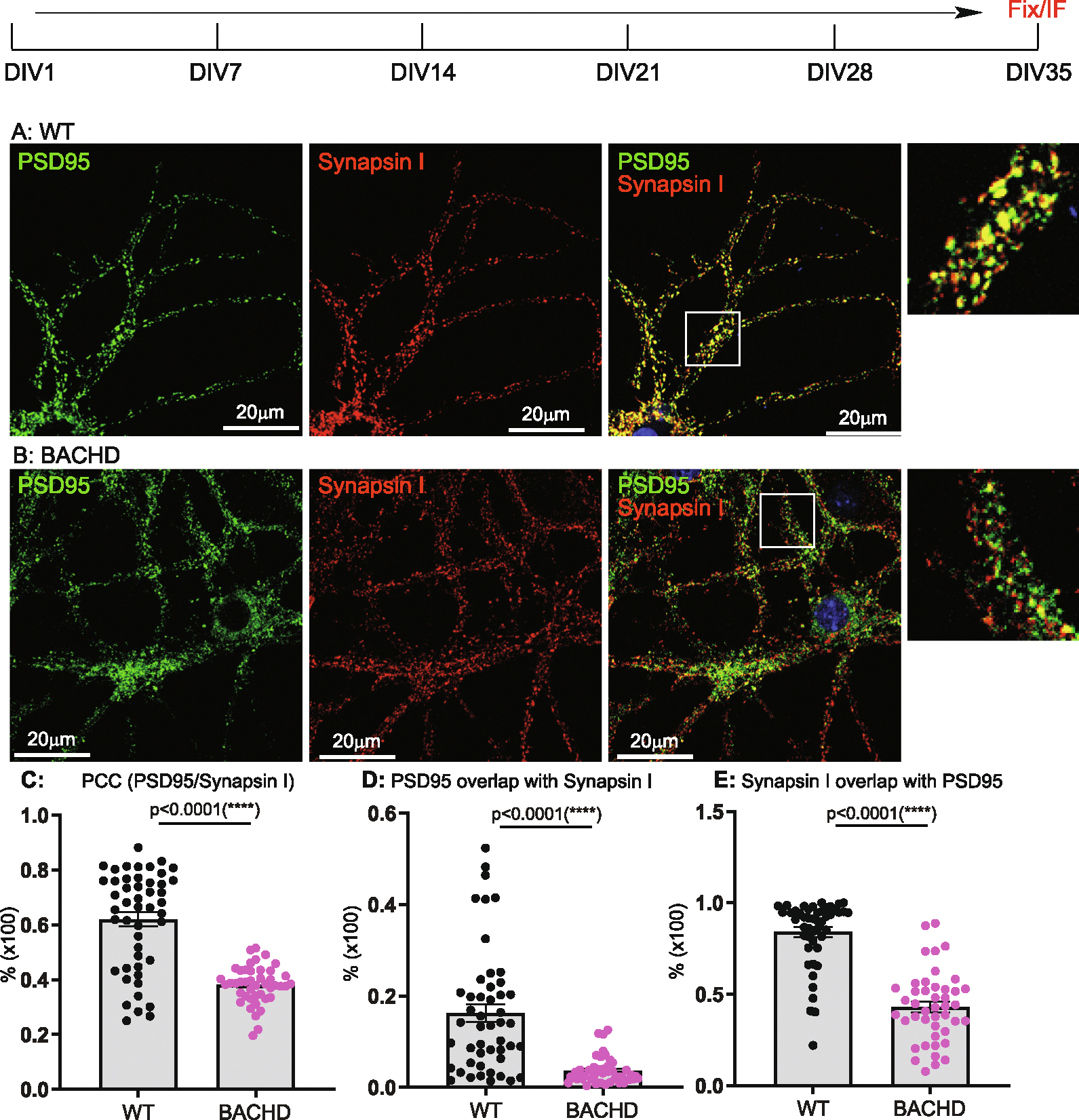
Synaptic analysis of cortical neurons from WT, BACHD at DIV35. Neuronal culture, immunostaining and quantitation are as described in Fig. 1. Representative images are shown and co-localization between synapsin I and PSD95 was quantitated using ImageJ Suite Plugin. Representative images of PSD95 (green) and Synapsin I (red) staining respectively in WT (**A**), BACHD (**B**) cortical neurons. Regions of interest marked by white boxes are magnified and shown on the right. **C**: Comparison of post- and presynaptic marker colocalization using PCC. **D**: Analysis of the % of PSD95/Synapsin I. **E**: Analysis of the % of Synapsin I/PSD95. Results are shown as mean ± SEM. The numbers of images were analyzed: *n* = 48 (WT), *n* = 44 (BACHD). The data represents at least 4–5 independent cultures. Significance analysis was carried out using Prism. Statistical significances were calculated by Sidak’s multiple comparisons test of One-Way ANOVA. n.s. = non significance. All p values are shown.

**Fig. 5.**
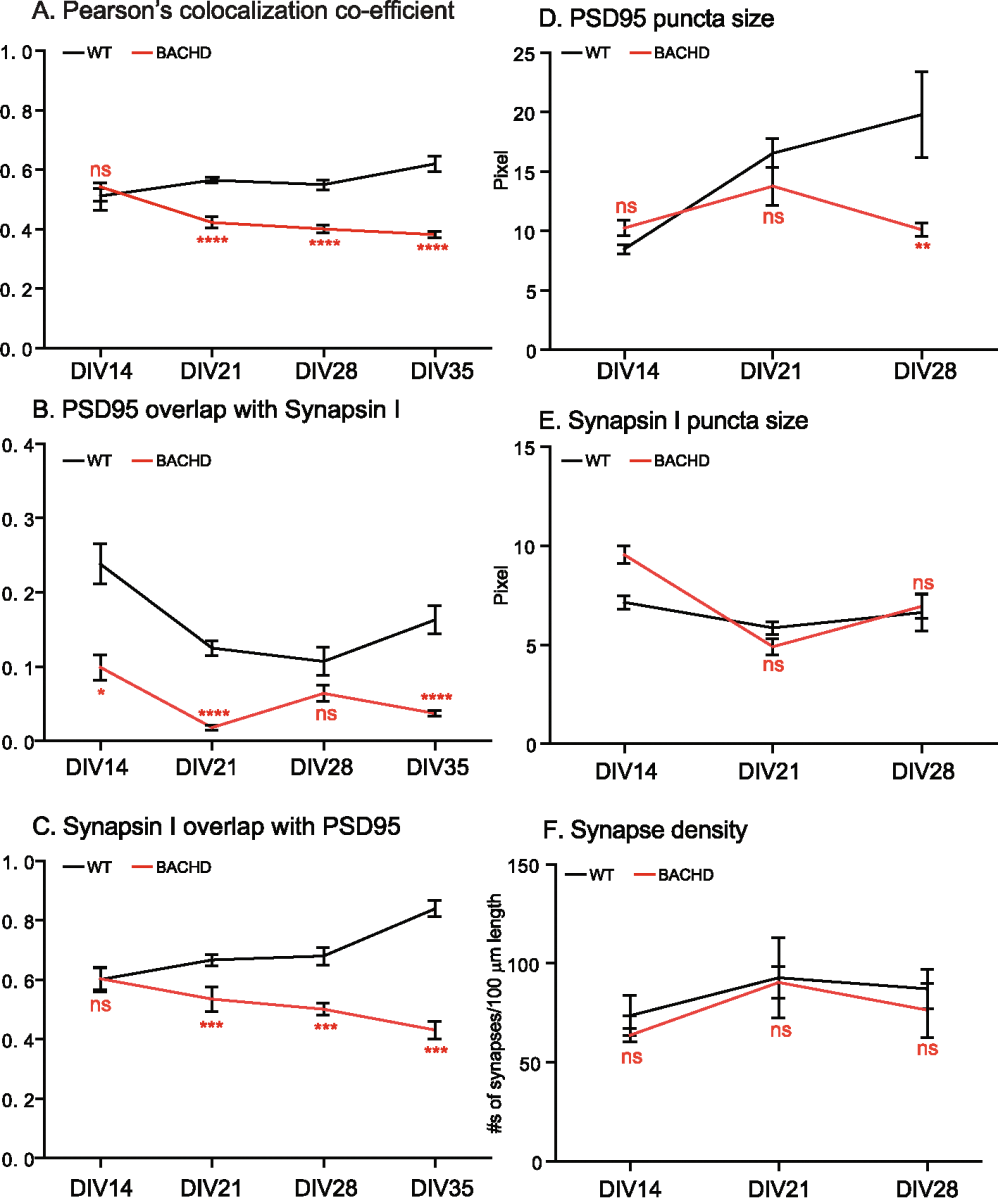
Time course of synaptic formation of cortical neurons from WT, BACHD from DIV14-DIV35. A time course study of Pearson’s colocalization co-efficient values from DIV14 to DIV 35 (in Fig. 1–4) was plotted to show the progression of synaptic formation in WT, BACHD cortical neurons. **A**: Time-dependent changes of PCC. **B**: Time-dependent changes of the % of PSD95/Synapsin I. **C**: Time-dependent changes of the % of Synapsin I/PSD95. **D**: Measurements for PSD95 puncta size in WT, BACHD cortical neurons from DIV14 to DIV28. **E**: Measurements for Synapsin I puncta size in two genotypes from DIV14-DIV28. **F**: Measurements for synaptic density in two genotypes from DIV14–28. Significance analysis was carried out using Prism. Statistical significances were calculated by One-Way ANOVA. n.s. = non significance. *P < 0.05, **P < 0.01, ***P < 0.001, ****P < 0.0001.

DIV14, 21, 28, 35 cultures were analyzed for synaptogenesis to report on the combined contributions of synapse formation and maintenance. Synapses were quantified by immunostaining with antibodies against Synapsin I for pre-synapses and against postsynaptic density 95 (PSD95) for post-synapses. The results were analyzed as described in the Materials and Methods. We used Pearson’s Colocalization Co-efficient (PCC) to quantitate synapses, as PCC measures the strength of a linear association between the distribution of PSD95 vs Synapsin I signals. We quantitated the percentage of post- and pre- synaptic staining that contributed to the synapses, as revealed by assessing the extent to which PSD95 signals overlapped with Synapsin I (i.e. % of PSD95/Synapsin I), and conversely, Synapsin I signals that overlapped with PSD95 (% of SynapsinI/PSD95). Representative images for DIV14 through DIV35 and corresponding quantitative results are presented in Figs. 1 to 4, respectively. All the data with accompanying *p* values are presented in Table 1 and presented in Fig. 5 (A–C). In addition, we measured the size of Synapsin1 and PSD95 puncta and synapse density (Fig. 5D–F, Table 2).

### 3.2. Colocalization of PSD95/Synapsin I in BACHD differs from WT only after DIV14

Synapses were readily evident by DIV14 in WT, BACHD cultures, a finding consistent with reports demonstrating that neurons form synapses at DIV6–7, with fully functional synapses appearing at DIV12–14 (Kavalali et al., 1999; Nosyreva et al., 2013). Analysis of synapses of WT neurons from DIV14–35 (Fig. 5A, Table 1) showed the PCC value was stable from DIV14 (Fig. 1A, C) to DIV21 (Fig. 2A, C) and to DIV28 (Fig. 3A, C), except for a slight increase at DIV35(Fig. 4A, C) (0.620 ± 0.026 from 0.511 ± 0.024, p < 0.05; One-Way ANOVA with Dunnett’s multiple comparison test).

The PCC value of BACHD cortical neurons was equivalent to WT neurons at DIV14 (Fig. 1B, C, Fig. 5A, Table 1). However, by DIV21 (Fig. 2B, C), the value decreased, with further smaller decreases at DIV28 (Fig. 3B, C) and DIV35 (Fig. 4B, C). Relative to WT at DIV14, the PCC values in BACHD neurons at all subsequent DIVs were statistically significantly different (*p* < 0.0001). The changes in PCC for WT, BACHD neurons from DIV14–35 are plotted in Fig. 5A (Also See Table 1). Overall, the PCC values of BACHD cortical neurons were reduced relative to WT from DIV21 onward (Fig. 5A). These results suggest that synaptogenesis in BACHD neurons likely is not impacted initially, but rather these neurons develop deficits in synapse maintenance.

### 3.3. Additional synaptic parameters distinguish BACHD from WT neurons

To further analyze changes in synapses in BACHD neurons, we quantitated the percentage of PSD95 signals colocalized with Synapsin I (% of PSD 95/Synapsin I) and vice versa (% of Synapsin I/PSD95) in the same cultures. With respect to the % of PSD95/Synapsin I (Table 1, Fig. 5B), WT neurons showed a significant decrease (*p* < 0.0001) from DIV14 (Fig. 1D) to DIV21 (Fig. 2D). The values at DIV28 (Fig. 3D) and DIV35 (Fig. 4D) were also significantly lower than for DIV14 (*p* = 0.001 for DIV28, *p* = 0.0135 for DIV35). For BACHD neurons, there was a decrease from DIV14 (Fig. 1D) to DIV21 (Fig. 2D). The values at DIV28 (Fig. 3D) and DIV35 (Fig. 4D) were higher than at DIV21 but still significantly (*p* = 0.026) lower than for DIV14. When compared to WT neurons, BACHD neurons showed a significant decrease in the % of PSD95/Synapsin I at DIV14 (*p* = 0.0002), DIV21(*P* < 0.0001), and DIV35 (P < 0.0001) and a strong trend to a decrease at DIV28 (*p* = 0.053) (Fig. 5B, Table 1). The overall reduction over time in the % of PSD95/Synapsin I in BACHD cultures was significant (p < 0.0001).

For the % of Synapsin I/PSD95 (Table 1, Fig. 5C), we saw a gradual increase in WT neurons from DIV14 (Fig. 1E) to DIV21 (Fig. 2E) and to DIV28 (Fig. 3E), with a significant increase at DIV35 (p < 0.0001) (Fig. 4E). In contrast, BACHD neurons showed a progressive decrease from DIV14 to DIV35 (Fig. 1E, 2E,3E 4E, 5C). Although not different from WT neurons at DIV14 (*p* = 0.955), BACHD neurons showed a significant reduction in the % of Synapsin I/PSD95 at DIV21 (*p* = 0.0017), DIV28 (p <0.0001), and DIV35 (p <0.0001) (Fig. 5C, Table 1). The reduction in BACHD versus WT neurons in the % of Synapsin I/PSD95 over time was highly significant (*p* = 0.0092) (Fig. 5C).

The changes in the % of either PSD95/Synapsin I or Synapsin I/PSD95 could reflect changes at the pre-, post-synapses, or both. To differentiate between these possibilities, we measured the sizes of PSD95 and Synapsin I puncta as well as synapse density from DIV14–28 (Table 2). Compared to WT neurons, the size of PSD95 puncta in BACHD neurons did not differ at DIV14 (*p* = 0.0722), or DIV21(*p* = 0.3744), but was significantly reduced at DIV28 (*p* = 0.0013) (Fig. 5D, Table 2). The size of BACHD Synapsin I puncta was larger at DIV14 (p = 0.001) but did not differ from WT cultures thereafter (Fig. 5E and Table 2). Our measurement of synapse density in neurites (per 100 μm length) did not reveal significant differences between WT and BACHD neurons at DIV14 (*p* = 0.5591), DIV21 (*p* = 0.9885) or DIV 28 (*p* = 0.5693) (Fig. 5F, Table 2). Taken together, the data points to differences between WT and BACHD neurons in several cortical synaptic parameters. They suggest involvement of both the presynaptic and postsynaptic compartments, especially the latter, as contributing to the decrease in synapse maintenance in BACHD cultures.

### 3.4. Changes in synapses in BACHD and WT neurons are related to culture age

We also measured culture-age dependent changes in synaptic metrics for both WT and BACHD neurons (Table 3). For both WT and BACHD, we compared the measurements at different DIVs against DIV14 by One Way ANOVA with Dunnett’s post-tests. For WT neurons, the PCC values were not significantly different from DIV14 at DIV21 (*p* = 0.1435) or 28 (*p* = 0.6552) but showed a significant increase at DIV35 (p = 0.001). However, the % of PSD95/Synapsin I was significantly reduced at DIV21 (*p* < 0.0001), DIV28 (p = 0.001), and DIV35 (p = 0.0135). Changes in the % of Synapsin I/PSD95 tracked the pattern for PCC - i.e. no significant difference at DIV21 (*p* = 0.2084), and DIV28 (*p* = 0.3367) with a significant increase at DIV35 (*p* < 0.0001). The PSD95 puncta size showed a significant increase at both DIV21 (*p* < 0.0001) and DIV28 (p < 0.0001). Synapsin I puncta size was reduced significantly at DIV21 (p = 0.02) but not at DIV28 (*p* = 0.7412). Synapse density did not differ either at DIV21 (*p* = 0.5668) or at DIV28 (*p* = 0.7396). The pattern of changes is consistent with increasing maturation of synapses over time in WT cultures and with increasing overlap of Synapsin1 relative to PSD95.

For BACHD neurons, the PCC values were significantly reduced at all DIVs when compared to DIV14: DIV21 (*p* < 0.0001), DIV28 (p < 0.0001), DIV35 (p < 0.0001). The % of PSD95/Synapsin I was also significantly reduced at all DIVs: DIV21 (p < 0.0001), DIV28 (p = 0.026), DIV35 (p < 0.0001). Changes in the % of Synapsin I/PSD95 showed no significant difference at DIV21 (*p* = 0.3839), or DIV28 (*p* = 0.1279), and a significant decrease at DIV35 (*p* = 0.0039). PSD95 puncta size showed a significant increase at DIV21 (*p* = 0.0379) but no difference at DIV28 (*p* = 0.992). Synapsin I puncta size was reduced significantly both at DIV21 (p < 0.0001) and at DIV28 (*p* = 0.0006). The measurement for synapse density did not differ from DIV14 either at DIV21 (*p* = 0.1525) or at DIV28 (p = 0.566). These results are evidence that BACHD cultures exhibit a progressive loss of ability for synaptic maintenance accompanying with changes in both presynaptic and postsynaptic markers.

### 3.5. BACHD cultures develop age-related reduction in synaptic proteins

The significant reduction of synapses in BACHD with culture age raised the possibility that synaptic proteins were reduced. To test this possibility, we carried out Western blotting analysis of primary neuronal cultures. E18 cortical neurons from WT and BACHD mice were cultured as described above. Protein lysates were assayed by immunoblotting with antibodies specific for synaptic proteins involved in SNARE complex formation (Synaptobrevin, SNAP25), calcium sensing (Synaptotagmin), synaptic vesicles (synaptophysin) and the post-synapse (PSD95) (Fig. S1). We chose to examine DIV21 and DIV28 cultures to examine synaptic proteins as it was between these ages that BACHD neurons showed significant differences from WT neurons in synapse number. Remarkably, at DIV21, BACHD neurons showed statistically significant increases in all proteins examined (Fig. S1). However, at DIV28, the levels of all these proteins were significantly decreased (Fig. S1). The latter is consistent with but exceeds in magnitude the reduction in synapse numbers at DIV28. These data are evidence for culture age-related changes in synaptic proteins in BACHD cultures. However, they do not demonstrate a linear relationship between synaptic protein levels and synaptogenesis in BACHD neurons.

### 3.6. BDNF secretion is significantly reduced in BACHD cortical neurons at DIV21

BDNF plays an important role in synaptogenesis and synaptic function (Park and Poo, 2013). Previously, we showed that anterograde axonal transport of BDNF was reduced in BACHD cortical axons (Zhao et al., 2016), suggesting the possibility that BACHD cortical neurons likely secreted less BDNF. To test directly this possibility, we collected conditioned media from WT and BACHD cortical neurons at DIV14 and DIV21 and measured BDNF by ELISA. The amount of secreted BDNF in each sample was normalized against total cell protein. At DIV14 the level of released BDNF in BACHD cultures did not differ significantly from WT neurons (*p* = 0.6627) (Fig. S2). However, at DIV21 BDNF released was significantly lower in BACHD cultures than in WT neurons (*p* = 0.0189) (Fig. S2). Though the differences between WT and BACHD were significant, the changes between DIV14 and DIV21 were not significant for either WT or BACHD neurons (*p* = 0.5123). We conclude that BDNF secretion from BACHD neurons is decreased with increased age. These data suggest that decreased BDNF release from BACHD cortical axons as possibly contributing to the failure in synapse maintenance.

### 3.7. BDNF treatment prevents/rescues synaptic deficits in BACHD cortical neurons

**Fig. 6.**
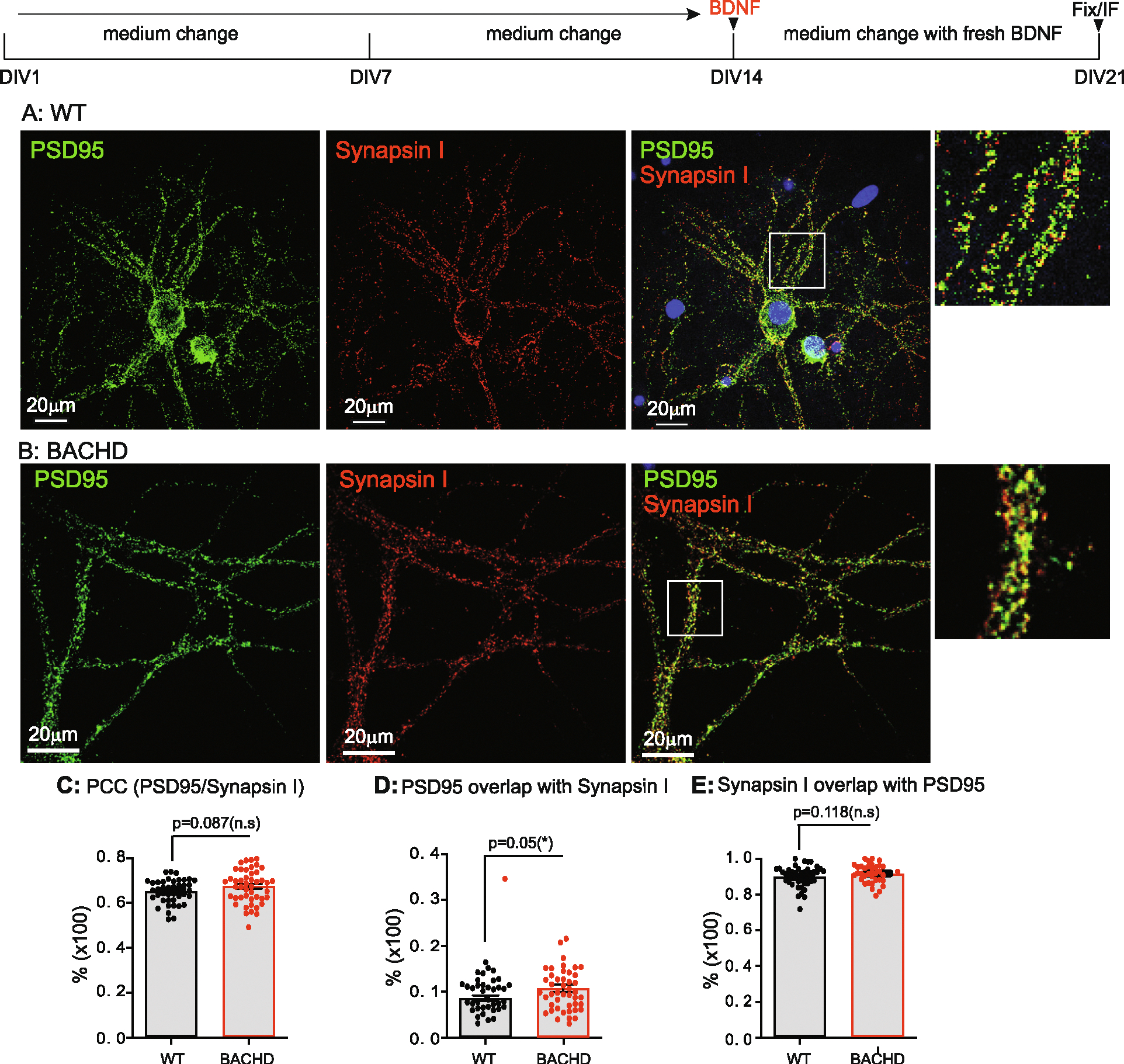
Rescuing effect of synaptic deficits in BACHD neurons by a 7-day treatment with BDNF. E18 cortical neurons from WT and BACHD were dis*sec*ted, cultured as in Fig. 1–4. Starting at DIV14, 100 ng/ml BDNF was added to the maintenance media and the media were replaced every other day until DIV21. Neurons were then fixed, immune-stained and quantitated for Pearson’s colocalization co-efficient as for Fig. 1–4. Representative images of WT(**A**) and BACHD (**B**) neurons stained for PSD95 (green) and Synapsin I (red). Regions of interest marked by white boxes are magnified and shown on the right. **C**: Comparison of post- and presynaptic marker colocalization using PCC. **D**: Analysis of the % of PSD95/Synapsin I. **E**: Analysis of the % of Synapsin I/PSD95. Results are shown as mean ± SEM. The numbers of images were analyzed for *n* = 42 (WT), *n* = 47 (BACHD). The data represents at least 4–5 independent cultures. Significance analysis was carried out using Prism. Statistical significances were calculated by unpaired Student’s t-test. n.s. = non significance. All p values are shown.

**Fig. 7.**
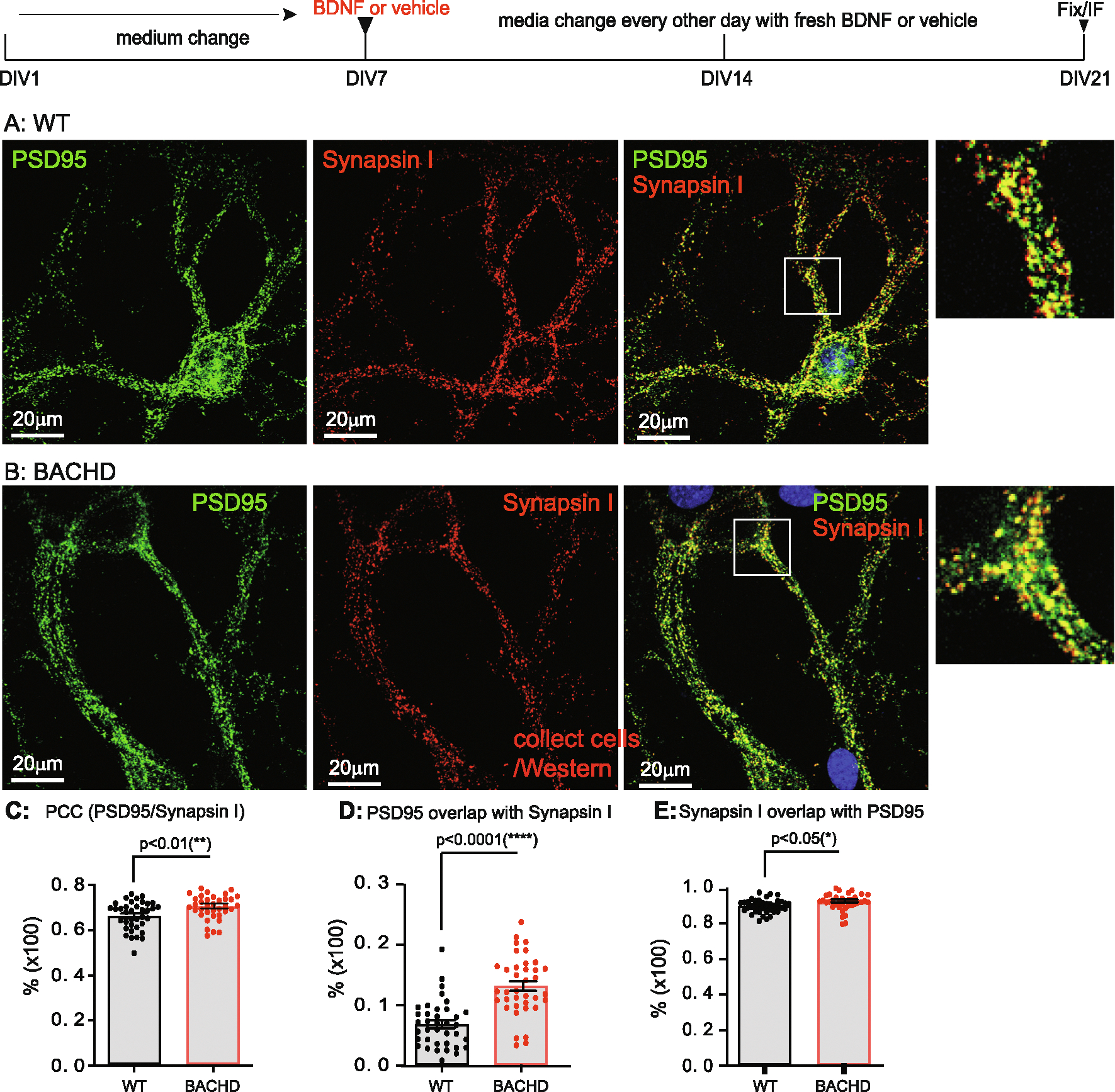
Rescuing effect of synaptic deficits in BACHD neurons by a 14-day treatment with BDNF. E18 cortical neurons from WT and BACHD were dissected, cultured as in Fig. 1–4. Starting at DIV7, 100 ng/ml BDNF was added to the maintenance media and the media were replaced every other day until DIV21. Neurons were then fixed, immune-stained and quantitated as in Fig. 6. Representative images of WT (**A**), BACHD (**B**) stained for PSD95 (green) and Synapsin I (red). Regions of interest marked by white boxes are magnified and shown on the right. C: Comparison of post- and presynaptic marker colocalization using PCC. **D**: Analysis of the % of PSD95/Synapsin I. **E**: Analysis of the % of Synapsin I/PSD95. Results are shown as mean ± SEM. The numbers of images were analyzed: *n* = 37 (WT), n = 37 (BACHD). The data represents at least 4–5 independent cultures. Significance analysis was carried out using Prism. Statistical significances were calculated by unpaired Student’s t-test. n.s. = non significance. All p values are shown.

To ask if addition of exogenous BDNF prevented synaptic deficits in BACHD neurons, we treated WT and BACHD cortical neurons with BDNF (50 ng/ml) for either 7 days (from DIV14 to DIV21) (Fig. 6) or 14-days (from DIV7 to DIV21) (Fig. 7). Cultures were immuno-stained for PSD95 and Synapsin I and PCCs (A, B in Figs. 6, 7) were computed as before. Both treatment regimens resulted in a significant increase in PCC - in WT (treated 7 days: p < 0.0001; treated 14 days: p < 0.0001) and BACHD neurons (7 days: p < 0.0001; 14 days: p < 0.0001) (Fig. 6C, 7C; Table 4). With BDNF treatment for 7 days (Fig. 6), the PCC value in BACHD neurons was comparable to that in WT neurons (*p* = 0.087) (Fig. 6C, Table 4). With a 14-day BDNF treatment (Fig. 7), PCC in BACHD neurons was increased further and was significantly higher than that in WT cultures (*p* < 0.01) (Fig. 7C, Table 4).

BDNF treatment also impacted other synaptic parameters. Following BDNF treatment, WT neurons showed a significant reduction in % of PSD95/Synapsin I in both 7-day treatments (*p* = 0.0038) and 14-day treatments (p < 0.0001) when compared to vehicle-treated samples (Table 4). In contrast, following BDNF treatment, BACHD neurons showed a significant increase in % of PSD95/Synapsin I in 7-day (p < 0.0001) and 14-day treatments (p < 0.0001) when compared to vehicle-treated samples (Table 4). The values were significantly greater than those in WT neurons for both 7-day (*p* = 0.05) (Fig. 6D) and 14-day treatments (p < 0.0001) (Fig. 7D).

BDNF treatment increased the % Synapsin I/PSD95 in both WT and BACHD neurons (Table 4). In WT cultures BDNF treatment increased % Synapsin I/PSD95 after both 7-day (*p* < 0.0038) and 14-day treatments (p < 0.0001) relative to vehicle-treated cultures (Table 4). In BACHD neurons, both 7-day (p < 0.0001) and 14-day treatments (p < 0.0001) of BDNF (Table 4) resulted in values that were equal to or greater than in WT neurons (Fig. 6E, 7E, Table 4). Therefore, consistent with earlier studies on its trophic effects on striatal neurons (Zhao et al., 2016), BACHD neurons responded to BDNF treatment with significant increases in synapse number and other parameters (i.e. PCC, %PSD95/Synapsin I and %Synapsin I/PSD95). The pattern of changes is consistent with a predominantly presynaptic effect in WT cultures with both pre- and postsynaptic actions in BACHD neurons (Table 4). That BACHD neurons were equally or more responsive than WT neurons is evidence of the continued ability of BDNF to act on BACHD cortical neurons to restore synaptic structure.

### 3.8. Synaptic deficits in BACHD cortical neurons are partially prevented by ApiCCT1

**Fig. 8.**
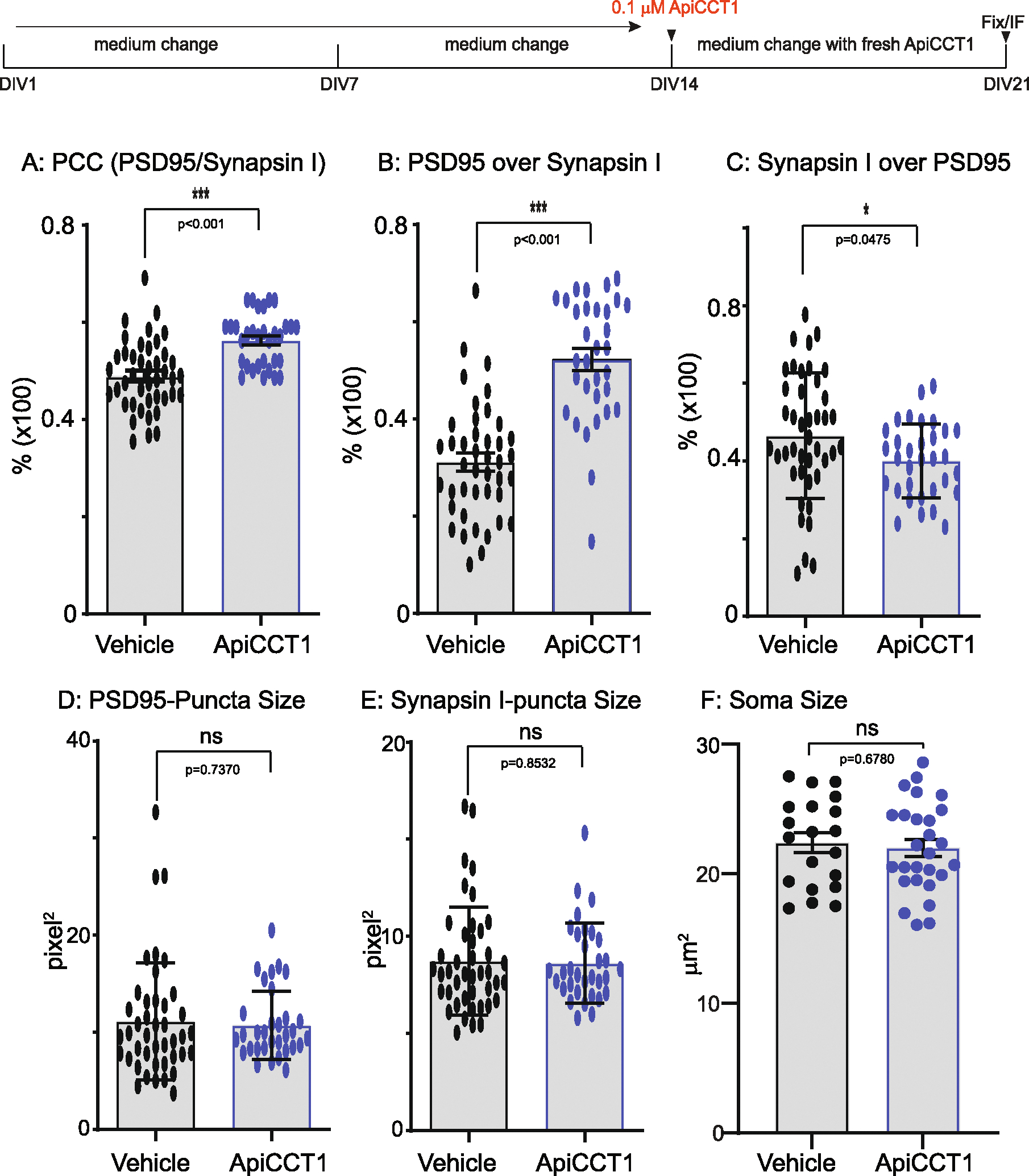
Rescuing effect of synaptic deficits in BACHD neurons by ApiCCT1. E18 cortical neurons from WT and BACHD were dissected, cultured as in Fig. 1–4. Starting at DIV14, 0.1 μM ApiCCT1 was added to the maintenance media and the media were replaced every other day until DIV21. Neurons were then fixed, immune-stained and quantitated as in Fig. 7. **A**: Comparison of post- and presynaptic marker colocalization using PCC in BACHD cultures with vehicle or ApiCCT1 treatment. **B**: Analysis of the % of PSD95/Synapsin I. **C**: Analysis of the % of Synapsin I/PSD95. **D**: Measurements of PSD95 puncta sizes. **E**: Measurements of Synapsin I puncta sizes. **F**: Size analysis of cortical neuronal soma in BACHD cultures treated with vehicle or ApiCCT1. Results are shown as mean ± SEM. The numbers of images were analyzed: n = 42 (WT), *n* = 32 (BACHD). The data represents at least 4–5 independent cultures. Significance analysis was carried out using Prism. Statistical significances were calculated by unpaired Student’s t-test. n.s. = non significance. All p values are shown.

Exogenous addition of ApiCCT1 rescued BDNF transport in BACHD cortical neurons (Zhao et al., 2016). To ask if ApiCCT1 treatment rescued synaptic deficits, we treated BACHD cortical neurons at DIV14 with either 0.1 μM ApiCCT1 or the vehicle control for 7 days. Synaptic staining and quantitation were carried out at DIV21. As compared to the vehicle, treatment with 0.1 μM ApiCCT1 significantly increased the PCC values (*p* <0.001) (Fig. 8A). The effect of ApiCCT1 was correlated with a significant increase in the % PSD95/Synapsin I (p < 0.001) (Fig. 8B); the % Synapsin I/PSD95 was modestly but significantly reduced (*p* = 0.048) (Fig. 8C). ApiCCT1 had no effect on puncta size for PSD95 (*p* = 0.74) (Fig. 8D), Synapsin I (*p* = 0.85) (Fig. 8E) or the size of soma (*p* = 0.6780) (Fig. 8F). We conclude that ApiCCT1 treatment partially prevented synaptic deficits in BACHD cortical neurons.

### 3.9. BACHD cortical neurons show significant functional deficits at DIV28 but not at DIV14

Since BACHD cortical neurons showed a culture-age dependent decrease in synapse maintenance, we next asked if these neurons were deficient in synaptic function. We used multielectrode arrays (MEA) to measure neuronal activities (Cotterill et al., 2016). The array contains a grid of tightly spaced electrodes embedded in the culture surface to record neuronal activity. When neurons fire action potentials, the extracellular voltage is measured by the electrodes on a microsecond timescale (Cotterill et al., 2016). MEA is well suited for measuring neuronal network activity and for sampling events at many locations across the culture to record initiation, propagation and synchronization of neural activity.

WT and BACHD cortical neurons were cultured in CytoView MEA 24-well plates pre-coated with poly-d-lysine (Axion Biosystems) at a density of 100,000 cells per well. At DIV14, neuronal activity and key features of neural network behaviors such as activity, synchrony, and network oscillations were recorded on the Maestro Edge (Axion) for 10 min. The data were batch processed using AxIS Navigator v2.0.4.21 and 12 metrics on neuronal activities were analyzed (for details, see Materials and Methods). As shown in Fig. S4, BACHD neurons at DIV14 showed no difference from WT (Fig. S3A–K) except for that IBI CoV showed a significant reduction denoting more regular network bursting (*p* < 0.05) (Fig.S3L). These functional findings complement those for synaptogenesis and synapse density at DIV14, that showed little difference between BACHD and WT neurons. We conclude that by and large at DIV14 BACHD cortical neurons form functional synapses with properties very similar to WT cultures at this stage.

**Fig. 9.**
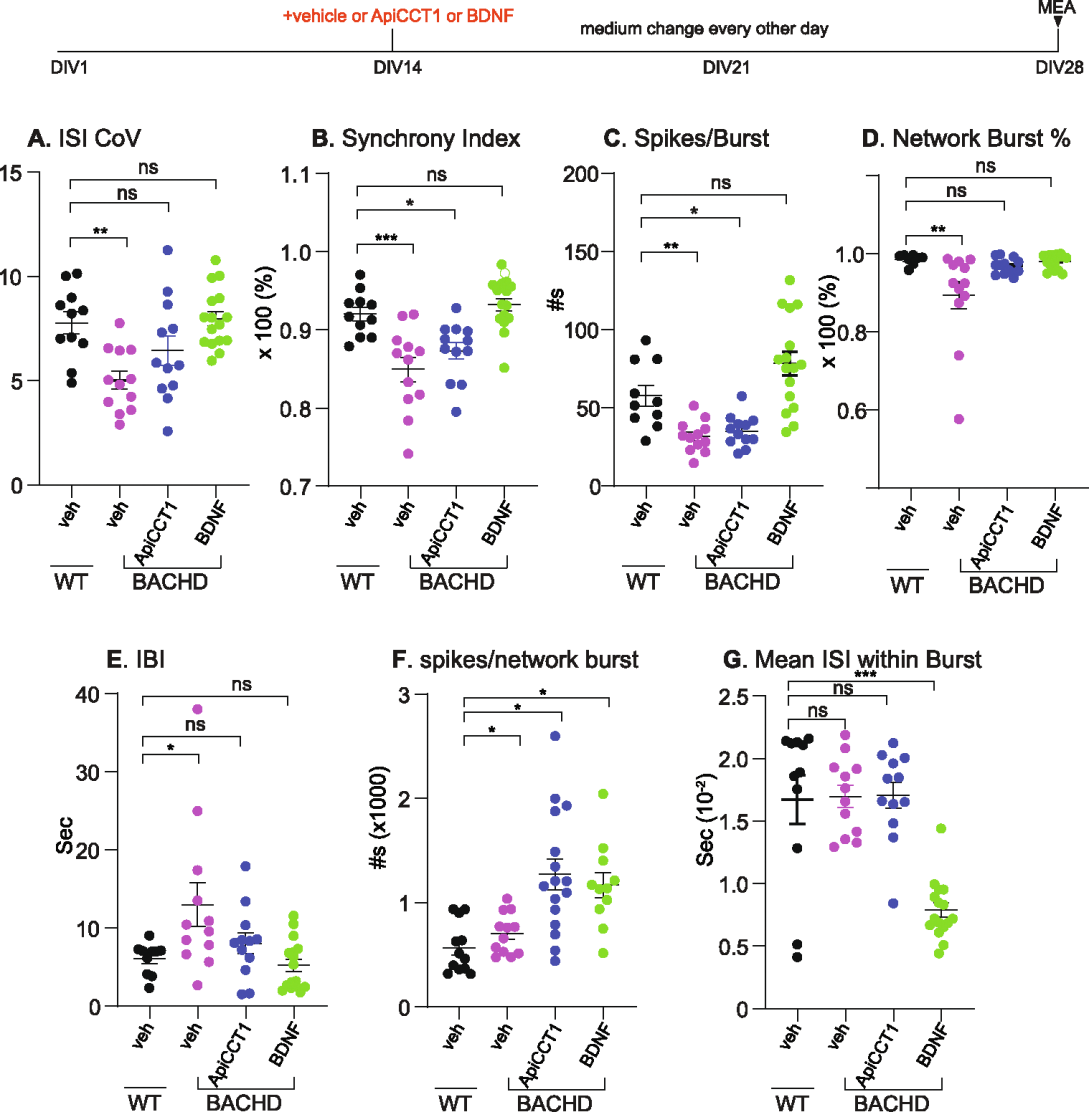
BACHD cortical neuronal activity shows significant deficits at DIV28. After completion of recording at DIV14, WT and BACHD neuronal cultures were treated with BDNF (50 ng/ml), ApiCCT1 (0.1 μM) or Vehicle. Media were changed every 48 h and a final recording was performed as above at DIV 28. Data were collected and quantitated as in Fig. 9: weighted mean firing rate (**A**), ISI (inter spike interval) coefficient of variation (**B**), the synchrony index (**C**), number of bursts (**D**), burst frequency (**E**), number of spikes/burst (**F**), inter burst interval (**G**), network burst% (**H**), number of spikes/network burst (**I**), mean ISI within burst, numbers of bursts (**J**), network bursts (**K**), network IBI CoV (**L**). Each data point represents one well of data. Analysis by two-way ANOVA. *: p < 0.05, **: *p* < 0.01, ***: *p* < 0.001, ****: p < 0.0001, ns = not significant.

At DIV28, several changes in synapse function characterized BACHD cultures with respect to WT cultures (Fig. 9). BACHD neurons showed significant changes; a significant decrease in: ISI CoV (Fig. 9A); 2) synchrony index (Fig. 9B); 3) spikes/burst (Fig. 9C); network burst percentage (Fig. 9D). An increase was seen for both IBI (Fig. 10E) and numbers of spikes/network burst (Fig. 9F). No significant deficits were observed either in Mean ISI within Burst (Fig. 9G), or in the following metrics: wMFR (Fig. S5 A), numbers of burst (Fig.S5B); burst frequency (Fig. S5C), network IBI COV (Fig.S5D), network bursts (Fig.S5E). The changes point to dysregulation of synaptic activity with differences in timing of spikes and bursts, the percentage of bursts and network synchrony.

Table 5 compares the findings at DIV28 to those at DIV14 for both WT and BACHD. Note that while WT cultures showed changes in only ISI CoV and network IBI CoV between DIV14 and DIV28, most electrophysiological parameters were significantly changed in BACHD cultures during this period reflecting an overall decrease in activity. As a result, WT and BACHD cultures showed marked differences at DIV28.

### 3.10. Deficits in BACHD neuronal activities are mitigated by BDNF and ApiCCT1

Given that BDNF and ApiCCT1 treatment of BACHD cultures positively impacted synapse maintenance and structure, we asked if these treatments would prevent changes in synaptic activity at DIV28. For these studies, treatment with BDNF or vehicle was initiated at DIV14 and the cultures were examined by MEA at DIV28. Representative raster plots depict the activities from all the electrodes in the well, providing important insights into the behavior of the culture (Fig. S4). In vehicle treated cultures, the amplitudes of BACHD activity were reduced and the pattern was irregular as compared to WT neurons (Fig. S4, B vs A). These changes were rescued with BDNF treatment (Fig. S4, D vs B), while BDNF showed no obvious effect in activity pattern for WT neurons (Fig. S4, C vs A). These results demonstrate a robust effect of BDNF treatment in normalizing neuronal activity in BACHD neurons.

Our detailed analysis of the MEA data reveals that BDNF treatment of BACHD neurons effectively normalized the deficits in ISI CoV (Fig. 9A), 2) synchrony index (Fig. 9B), 3) spikes/burst (Fig. 9C), network burst percentage (Fig. 9D) and IBI (Fig. 9E). Interestingly, BDNF seemed to induce an additional increase in spikes/network burst (Fig. 9F) while causing a significant reduction in the mean ISI within burst (Fig. 9G). BDNF did not alter measurements in 5 metrics (wMFR, numbers of burst, burst frequency, network IBI COV, network bursts (Fig. S5 A–E).

To examine further the effects of BDNF treatment, and to specifically address the effects within the same genotype, we also used One-Way ANOVA to examine WT and BACHD synaptic activity (Table 6). Among the 12 MEA metrics measured, when compared to vehicle treat, BDNF treatment of WT neurons induced a significant change only by reducing mean ISI within burst. However, treatment of BACHD neurons with BDNF significantly impacted many MEA metrics (Table 6): 1) mWFR; 2) ISI CoV; 3) Synchrony index; 4) spikes/burst; 5) IBI; 6) Network burst %; 7) Spikes/network burst; and 8) Mean ISI within burst. The data are evidence that BACHD neurons are robustly responsive to BDNF treatment with respect to synaptic function such that several deficits are rescued, and additional parameters are enhanced to the same extent as in BDNF treated WT neurons.

ApiCCT1 treatment also showed a rescuing effect in ISI COV (Fig. 9A), network burst % (Fig. 9 D) and IBI (Fig. 9E). As BDNF, ApiCCT1 also induced an additional increase in spikes/network burst (Fig. 9F). No effect for ApiCCT1 was seen in all other MEA metrics (Fig. 9, and Fig. S5). While there was no apparent ApiCCT1 effect on WT cultures using Two-Way ANOVA, analysis using One-Way ANOVA to emphasize effects within the same genotype (Table 6) showed that ApiCCT1 treatment of WT neurons induced significant changes in: 1) numbers of bursts; 2) Burst frequency; 3) IBI; and 4) Network bursts. These findings point to different treatment efficacies on synaptic parameters for BDNF and ApiCCT1 at the concentrations tested on BACHD and WT neurons.

Based on these findings, we conclude that BDNF treatment normalized BACHD-mediated synaptic changes and increased additional parameters reflecting synaptic function while ApiCCT1 only showed a partial effect.

## 4. Discussion

In our present study, we demonstrate that BACHD cortical neurons develop synaptic dysfunction. Although they do form functional synapses at early stages in vitro, BACHD neurons show significant deficits in synapse maintenance at more advanced culture ages. We further show that BDNF treatment effectively rescues these deficits while ApiCCT1 induces similar but less robust effects, especially with respect to synaptic function. Together with other studies (Buren et al., 2016; S. Chen et al., 2018; Mackay et al., 2023; Schmidt et al., 2018; Virlogeux et al., 2018), our findings demonstrate the ability to explore and define, in vitro, synaptic deficits that may underlie important mechanisms of HD pathogenesis.

In HD, synaptic dysfunction represents one of the most sensitive measures of disease pathogenesis (Barron et al., 2021; Cummings et al., 2007; Lepeta et al., 2016; Li et al., 2003; Milnerwood et al., 2006; Milnerwood and Raymond, 2007; Murphy et al., 2000; Nithianantharajah and Hannan, 2013; Taoufik et al., 2018). We thus established a long-term culture system of cortical neurons to track synaptogenesis, synaptic maintenance and synaptic function. Our results demonstrated that, although BACHD cortical neurons formed functional synapses at DIV14 that in most ways were equivalent to WT neurons, they showed progressive impairments in synapse maintenance and activity thereafter (DIV21–35). Of note, even though synaptogenesis as measured by PCC was normal for BACHD neurons at DIV14, the actual percentage of overlap between PSD95 and Synapsin I was different, indicating that at this time synapses were already different. Thus, it seems that mHTT was already at work at this early phase of synapse formation/maintenance. Nevertheless, in line with the synapse formation results, BACHD neurons did not exhibit overt functional deficits at DIV14. Significant changes in MEA metrics were detected only after later stages e.g. DIV28.

Deficits in synaptic maintenance and synaptic function in BACHD neurons is likely linked to the reduced ability by which these neurons secret BDNF (Zhao et al., 2016), as we demonstrated that the level of BDNF in conditioned media of BACHD neurons was significantly reduced at DIV21, but not at DIV14, a finding closely tracking the changes in the deficits of synaptic maintenance and neuronal activity. BDNF plays an important role in regulating synapse formation and function(Lu et al., 2014; Nithianantharajah and Hannan, 2013; Santos et al., 2010; Yoshii and Constantine-Paton, 2010). Although reduced BDNF supply from cortical neurons has been implicated as contributing to striatal degeneration in HD (Baydyuk and Xu, 2014; Gauthier et al., 2004; Su et al., 2014; Yu et al., 2018; Zuccato and Cattaneo, 2007, 2009), our current study has revealed that reduced BDNF is also likely responsible for synaptic deficits in BACHD cortical neurons.

Accordingly, our current study provides evidence that BDNF has a robust rescuing effect on synaptic dysfunction in BACHD cortical neurons by normalizing of the age-related deficits in neuronal activities. As for rescuing both synapse maintenance and neuronal activity. ApiCCT1 at the concentration tested here (0.1 μM) was much less effective than BDNF. It is likely that higher concentrations of ApiCCT1 are needed to achieve better efficacy, since 1.0 μM of ApiCCT1 was used in our previous study in demonstrating its rescuing effect on axonal transport of BDNF (Zhao et al., 2016). We conclude that BDNF robustly acted to enhance synaptic structure and function in BACHD neurons. Indeed, BDNF exerted effects on some measures in BACHD neurons that appear to have somewhat exceeded those in BDNF-treated WT neurons. However, some caveats apply for the interpretation of BDNF effects at synapses. One is that it is likely that many of the parameters measured by MEA are linked to BDNF effects on other cellular functions that indirectly support synaptic structure and function. Another one is that the amounts of BDNF (50 ng/ml) used in our studies almost certainly exceeded the physiological levels present at synapses in vivo. Additional study is needed to determine the minimal level of BDNF required for rescuing synapses and the mechanisms responsible.

Our current study is consistent with the idea that BDNF effects on neurons are widespread and extend from changes in gene expression to protein function. (Baydyuk and Xu, 2014; Lu et al., 2014; Moya-Alvarado et al., 2023; Santos et al., 2010; Su et al., 2014). How BDNF acts to restore synapses is an important question. Previous studies have shown that decreased BDNF-TrkB signaling led to reduction in the density of striatal dendritic spines in both the BACHD and the Q175 knock-in mouse models of HD(Plotkin et al., 2014). BDNF overexpression in the forebrain effectively restored dendritic spines density and morphology in striatal neurons of an HD mouse model(Xie et al., 2010). Furthermore, immunohistochemical staining against pre-synaptic (VGLUT1) and post-synaptic (PSD95) markers showed that the decrease of cortico-striatal synapses was significantly improved by increased expression of BDNF in HD models(Giralt et al., 2011).

In conclusion, our in vitro studies have demonstrated significant age-dependent synaptic deficits in BACHD neurons. These deficits correlate with reduced release of BDNF that are effectively restored by exogenous BDNF. The culture paradigm we employed can be used to further explore HD synaptic pathogenesis and treatments to intercept synaptic pathology in HD (Buren et al., 2016; Mackay et al., 2023; Schmidt et al., 2018; Virlogeux et al., 2018).

## Supplementary Material

Supplement

## Figures and Tables

**Table 1:** Analysis of synaptic formation in cultured neurons of WT, BACHD (I).

	PCC		% of PSD95/Synapsin I		% of Synapsin I/PSD95	
			
	WT	BACHD	WT	BACHD	WT	BACHD

DIV14	0.511 ± 0.024	0.542 ± 0.014^ns^ *p* = 0.326	0.238 ± 0.027	0.099 ± 0.017*** p = 0.0002	0.601 ± 0.042	0.604 ± 0.036^ns^ p = 0.955
DIV21	0.565 ± 0.010	0.422 ± 0.019**** p < 0.0001	0.125 ± 0.010	0.018 ± 0.003**** p < 0.0001	0.667 ± 0.019	0.535 ± 0.041**** p = 0.0017
DIV28	0.549 ± 0.016	0.4 ± 0.012**** p < 0.0001	0.107 ± 0.019	0.064 ± 0.011^ns^ p = 0.053	0.68 ± 0.03	0.501 ± 0.02**** p < 0.0001
DIV35	0.620 ± 0.026	0.381 ± 0.01**** p < 0.0001	0.163 ± 0.019	0.037 ± 0.004**** p < 0.0001	0.84 ± 0.026	0.431 ± 0.029**** p < 0.0001

Significance test: One Way ANOVA (Dunnett’s post-tests: BACHD vs WT): ns = non-significance, *p* > 0.05;

* *p* < 0.05;

** *p* < 0.01;

*** *p* < 0.001;

**** *p* < 0.0001.

**Table 2:** Analysis of synaptic formation in cultured neurons of WT, BACHD(II).

	DIV14		DIV21		DIV28	
	WT	BACHD	WT	BACHD	WT	BACHD

PSD95 (pixel)	8.48 ± 0.383	10.27 ± 0.677^ns^ p = 0.0722	16.56 ± 1.22	13.78 ± 1.6^ns^ p = 0.3744	19.9 ± 3.61	10.11 ± 0.59** p = 0.0013
Synapsin I (pixel)	7.14 ± 0.33	9.54 ± 0.45** p = 0.0001	5.85 ± 0.32	4.90 ± 0.41^ns^ *p* = 0.2389	6.64 ± 0.94	6.95 ± 0.6^ns^ *p* = 0.9343
#synapses/100 μm	73.68 ± 10.12	63.79 ± 3.51^ns^ p = 0.5591	92.82 ± 20.08	90.46 ± 7.94^ns^ p = 0.9885	87.15 ± 4.07	76.4 ± 5.69* p = 0.5693

Significance test: One Way ANOVA (Dunnett’s post-test). ns = non-significance, p > 0.05;

* p < 0.05;

** *p* < 0.01.

**Table 3:** Culture-age dependent changes in synaptic measurements.

Measurement	Comparison	WT		BACHD	
		significance	p value	significance	p value

	DIV21 vs DIV14	ns	0.1435	****	<0.0001
	DIV28 vs DIV14	ns	0.6552	****	<0.0001
PCC	DIV35 vs DIV14	**	0.001	****	<0.0001
	DIV21 vs DIV14	****	<0.0001	****	<0.0001
	DIV28 vs DIV14	***	0.001	*	0.026
PSD95/Synapsin I	DIV35 vs DIV14	*	0.0135	****	<0.0001
	DIV21 vs DIV14	ns	0.2084	ns	0.3839
	DIV28 vs DIV14	ns	0.3367	ns	0.1279
Synapsin I/PSD95	DIV35 vs DIV14	****	<0.0001	**	0.0039
	DIV21 vs DIV14	****	<0.0001	*	0.0379
PSD95 Puncta Size	DIV28 vs DIV14	****	<0.0001	ns	0.992
	DIV21 vs DIV14	*	0.02	****	<0.0001
Synapsin I Puncta Size	DIV28 vs DIV14	ns	0.7412	***	0.0006
	DIV21 vs DIV14	ns	0.5668	ns	0.1525
#synapses/100 μm	DIV28 vs DIV14	ns	0.7396	ns	0.566

Significance test: One Way ANOVA (Dunnett’s post-tests). ns = non-significance, p > 0.05;

* p < 0.05;

** p < 0.01;

*** p < 0.001),

**** p < 0.0001).

**Table 4:** Analysis of BDNF effects on synaptic formation in WT, BACHD.

	WT			BACHD		
	Vehicle	BDNF 7 d	BDNF14 d	Vehicle	BDNF 7d	BDNF 14d

PCC	0.565 ± 0.010	0.651 ± 0.008**** p < 0.0001	0.665 ± 0.010**** p < 0.0001	0.422 ± 0.019	0.675 ± 0.011**** p < 0.0001	0.705 ± 0.009**** p < 0.0001
PSD95/Synapsin I	0.125 ± 0.010	0.086 ± 0.006** p = 0.0038	0.069 ± 0.006**** p < 0.0001	0.018 ± 0.003	0.107 ± 0.008**** p < 0.0001	0.132 ± 0.008**** p < 0.0001
Synapsin I/PSD95	0.667 ± 0.019	0.900 ± 0.009**** p < 0.0038	0.896 ± 0.006**** p < 0.0001	0.535 ± 0.041	0.918 ± 0.007**** p < 0.0001	0.918 ± 0.008**** p < 0.0001

Significance test: One Way ANOVA (Dunnett’s post-tests: BDNF 7d vs Vehicle; BDNF 14 d vs vehicle). ns = non-significance, *p* > 0.05;

* *p* < 0.05;

** p < 0.01;

*** p < 0.001),

**** p < 0.0001);

**Table 5:** Comparison of neuronal activities.

	WT: DIV14 vs DIV28			BACHD: DIV14 vs DIV28			WT DIV28 vs BACHD DIV28		
MEA metrics	DIV14	DIV28	p value	DIV14	DIV28	p value	WT	BACHD	p value

A. wMFR (Hz)	13.38 ± 1.08	12.55 ± 1.79	0.3354(ns)	12.69 ± 1.01	5.72 ± 1.15	0.0027(**)	12.55 ± 1.79	5.72 ± 1.15	0.0015(**)
B. ISI CoV	5.51 ± 0.29	7.77 ± 0.52	0.0009 (***)	4.98 ± 0.17	5.02 ± 0.44	0.8898(ns)	7.77 ± 0.52	5.02 ± 0.44	0.0012(**)
C. Synchrony Index	0.88 ± 0.01	0.92 ± 0.01	0.3552(ns)	0.86 ± 0.02	0.85 ± 0.02	0.1574(ns)	0.92 ± 0.01	0.85 ± 0.02	0.0012(**)
D. #s of Bursts	3112 ± 324	2046 ± 242	0.2631(ns)	2839 ± 244	1425 ± 254	0.0117(*)	2046 ± 242	1425 ± 254	0.0337(*)
E. Burst Frequency (Hz)	0.33 ± 0.03	0.25 ± 0.03	0.2888(ns)	0.30 ± 0.03	0.15 ± 0.03	0.0117 (*)	0.25 ± 0.03	0.15 ± 0.03	0.0337(*)
F. Spikes/Burst	48.70 ± 2.70	58.73 ± 6.11	0.1582(ns)	44.55 ± 2.57	31.59 ± 2.90	0.026(*)	58.73 ± 6.11	31.59 ± 2.90	0.0012(**)
G. IBI (sec)	9.95 ± 2.18	6.44 ± 0.67	0.1001(ns)	9.52 ± 2.57	13.03 ± 2.83	0.0025 (**)	6.44 ± 0.67	13.03 ± 2.83	0.0289(ns)
H. Network Burst%	94.31 ± 1.17	98.35 ± 0.36	0.0886(ns)	93.76 ± 1.44	89.42 ± 3.50	0.0375 (*)	98.35 ± 0.36	89.42 ± 3.50	0.0028(ns)
I. Spikes/Network Burst	986 ± 43	1169 ± 121	0.0749(ns)	944 ± 43	566 ± 70	0.0004 (***)	1169 ± 121	566 ± 70	0.0005 (***)
J. Mean ISI w/n Burst	0.016 ± 0.001	0.017 ± 0.002	0.8916(ns)	0.017 ± 0.001	0.017 ± 0.001	0.9484(ns)	0.017 ± 0.002	0.017 ± 0.001	0.5181(ns)
K. Network Bursts	137 ± 13	108 ± 17	0.2351(ns)	137 ± 11	96 ± 19	0.0873(ns)	108 ± 17	96 ± 19	0.5795(ns)
L. Network IBI CoV	0.94 ± 0.07	0.64 ± 0.08	0.0286 (*)	0.73 ± 0.06	0.88 ± 0.15	0.5405(ns)	0.64 ± 0.08	0.88 ± 0.15	0.2549(ns)

Significance test: nonparametric t-test (Mann-Whitney). ns = non-significance, p > 0.05;

* p < 0.05;

** p < 0.01;

*** p < 0.001).

**Table 6:** Effects of ApiCCT1, BDNF on neuronal activities.

	WT DIV28			BACHD DIV28		
MEA metrics (DIV28)	Vehicle	ApiCCT1	BDNF	Vehicle	ApiCCT1	BDNF

A. wMFR (Hz)	12.55 ± 1.79	14.82 ± 2.22^ns^ *p* = 0.6572	19.24 ± 3.56^ns^ *p* = 0.13	5.72 ± 1.15	10.02 ± 3.08^ns^ *p* = 0.6119	22.97 ± 4.29*** p = 0.0008
B. ISI CoV	7.77 ± 0.52	6.23 ± 0.47^ns^ *p* = 0.1195	7.12 ± 0.60^ns^ *p* = 0.7451	5.02 ± 0.44	6.44 ± 0.71^ns^ *p* = 0.1087	7.99 ± 0.38*** p = 0.0003
C. Synchrony Index	0.92 ± 0.01	0.91 ± 0.01^ns^ *p* = 0.8628	0.92 ± 0.01^ns^ *p* = 0.9918	0.85 ± 0.02	0.87 ± 0.01^ns^ *p* = 0.268	0.93 ± 0.01**** p < 0.0001
D. #s of Bursts	2046 ± 242	3046 ± 352* p = 0.0162	2349 ± 127^ns^ *p* = 0.5729	1425 ± 254	2287 ± 565^ns^ *p* = 0.2814	2720 ± 372^ns^ *p* = 0.055
E. Burst Frequency (Hz)	0.25 ± 0.03	0.32 ± 0.04* p = 0.0173	0.25 ± 0.01^ns^ *p* = 0.5653	0.15 ± 0.03	0.24 ± 0.06^ns^ *p* = 0.2813	0.28 ± 0.04^ns^ p = 0.055
F. Spikes/Burst	58.73 ± 6.11	46.9 ± 7.75^ns^ *p* = 0.6952	81.2 ± 13.8^ns^ *p* = 0.2657	31.59 ± 2.90	35.3 ± 2.9^ns^ *p* = 0.8722	78.2 ± 7.39**** p < 0.0001
G. IBI (sec)	6.44 ± 0.67	4.65 ± 0.67* p = 0.0042	4.84 ± 0.21^ns^ *p* = 0.0547	13.03 ± 2.83	8.05 ± 1.32^ns^ *p* = 0.1035	5.25 ± 0.80** p = 0.0046
H. Network Burst%	98.35 ± 0.36	98.21 ± 0.47^ns^ *p* = 0.9279	96.79 ± 0.80^ns^ *p* = 0.1237	89.42 ± 3.50	96.9 ± 0.59* p = 0.02	98.04 ± 0.47** p = 0.004
I. Spikes/Network Burst	1169 ± 121	939.9 ± 187^ns^ *p* = 0.6651	1284 ± 232^ns^ *p* = 0.8849	566 ± 70	703 ± 56.4^ns^ *p* = 0.6228	1272 ± 147*** p = 0.0001
J. Mean ISI w/n Burst	0.017 ± 0.002	0.0146 ± 0.002^ns^ *p* = 0.5863	0.009 ± 0.001** p = 0.0045	0.017 ± 0.001	0.0170 ± 0.001^ns^ *p* = 0.996	0.008 ± 0.001**** p < 0.0001
K. Network Bursts	108 ± 17	174.9 ± 24.1* p = 0.0302	163.1 ± 12.4^ns^ p = 0.0605	96 ± 19	130.8 ± 33.8^ns^ *p* = 0.5803	174.9 ± 24.6^ns^ *p* = 0.0695
L. Network IBI CoV	0.64 ± 0.08	0.58 ± 0.09^ns^ *p* = 0.8852	0.59 ± 0.11^ns^ *p* = 0.8959	0.88 ± 0.15	0.84 ± 0.17^ns^ *p* = 0.9754	0.57 ± 0.07^ns^ *p* = 0.1592

Significance test (WT or BACHD: ApiCCT1 vs Vehicle; BACHD vs Vehicle): One Way ANOVA (Dunnett’s multi comparison) ns = non-significance p > 0.05;

* p < 0.05;

** p < 0.01;

*** p < 0.001).

## Data Availability

Data will be made available on request.
