# Supplementary material for: BDNF and TRiC-inspired reagent rescue cortical synaptic deficits in a mouse model of Huntington’s disease
Source: Neurobiol Dis. Author manuscript; Available in PMC 2025 Mar 9. (PMC11890210; doi:10.1016/j.nbd.2024.106502)
Supplement: Supplement [file NIHMS2057913-supplement-Supplement.pdf]

Supporting Materials

Supplemental Figures

Figure S1. Western analysis of changes in synaptic proteins.
WT and BACHD neurons were cultured and protein lysates were collected in RIPA buffer at DIV21 and DIV28. Protein lysates were analyzed by SDS-PAGE/immunoblotting with specific antibodies as indicated. Beta actin was used as a loading control. Exposures within linear range were quantitated using BioRad Image Lab 6.0 and are normalized against beta actin. Significance tests are performed in Prism (unpaired t-test). * $p<0.05$; ** $p<0.01$; *** $p<0.001$.

Figure S2. Measurement of secreted BDNF in conditioned media from cultured neurons
E18 cortical neurons from WT and BACHD were dissected, cultured on PLL-coated 12-well plates and maintained as described in Materials and Methods. Conditioned media were collected at DIVs and the amounts of BDNF were measured by ELISA as described in the Materials and Methods. Corresponding cell lysates were collected with total proteins measured using BCA. Secreted BDNF was normalized against the respective protein content of each sample. (A) Standard curve of the first measurement. (B) Comparison of BDNF secretion in conditioned media in WT and BACHD cortical cultures at DIV14 and DIV21. (C) Standard curve of the second measurement. (D) Same comparison as (B). Results are shown as mean ± SEM. Significance analysis was carried out using Prism. Statistical significances were calculated by unpaired Student's t test. n.s.= non significance. All p values are shown in the graphs.

Figure S3. BACHD cortical neuronal activity shows no significant deficits at DIV14.
E18 cortical neurons from WT and BACHD were plated on Poly-D-Lysine coated (100 µg/ml – Sigma Aldrich) CytoView MEA 24 well plates (Axion Biosystems) at a density of 100,000 cells per well. At DIV14, neuronal activity and key features of neural network behavior as functional endpoints - activity, synchrony, and network oscillations were recorded on the Maestro Edge (Axion) for 10 min. The data were batch processed using AxIS Navigator v2.0.4.21. E18 BACHD cortical neurons showed no difference from WT in weighted mean firing rate (**A**), ISI (inter spike interval) coefficient of variation (**B**), the synchrony index (**C**), number of bursts (**D**), burst frequency (**E**), number of spikes/burst (**F**), inter burst interval (**G**), network burst% (**H**), number of spikes/network burst (**I**), mean ISI within burst, numbers of bursts (**J**), network bursts (**K**), network IBI CoV (**L**). BACHD cortical neurons differ from WT neurons only in network IBI CoV (**L**). Each data point represents one well of data. Analysis by two-way ANOVA. * $p<0.05$, ns=not significant.

Figure S4. Rescuing effects of BDNF on BACHD synaptic activity at DIV28.
After completion of recording at DIV14, WT and BACHD neuronal cultures were treated with BDNF (50 ng/ml) or Vehicle. Media were changed every 48 hrs and a final recording was performed as above at DIV 28. MEA recording was performed as in Fig. 9, 10. Representative examples of neuronal activity bursting over 180 secs are presented. The spike histograms (top) and network bursts (marked by a purple box) are shown. A: WT neurons treated with vehicle; B: BACHD neurons treated with vehicle; C: WT neurons treated with BDNF; D. BACHD neurons treated with BDNF.

Figure S5. MEA metrics do not differ between BACHD and WT neurons and are not impacted by treatment at DIV28.
As in **Figure 9**, E18 cortical neurons from WT and BACHD were plated on Poly-D-Lysine coated (100 µg/ml – Sigma Aldrich) CytoView MEA 24 well plates (Axion Biosystems) at a density of 100,000 cells per well. At DIV14, neuronal activity and key features of neural network behavior as functional endpoints - activity, synchrony, and network oscillations were recorded on the Maestro Edge (Axion) for 10 min. The data were batch processed using AxIS Navigator v2.0.4.21. E18 BACHD cortical neurons showed no difference from WT in weighted mean firing rate (**A**), number of spikes/burst (**B**), burst frequency (**C**), network IBI CoV (**D**), network bursts (**E**). Each data point represents one well of data. Analysis by two-way ANOVA. * $p<0.05$, ns=not significant.

Fig S1

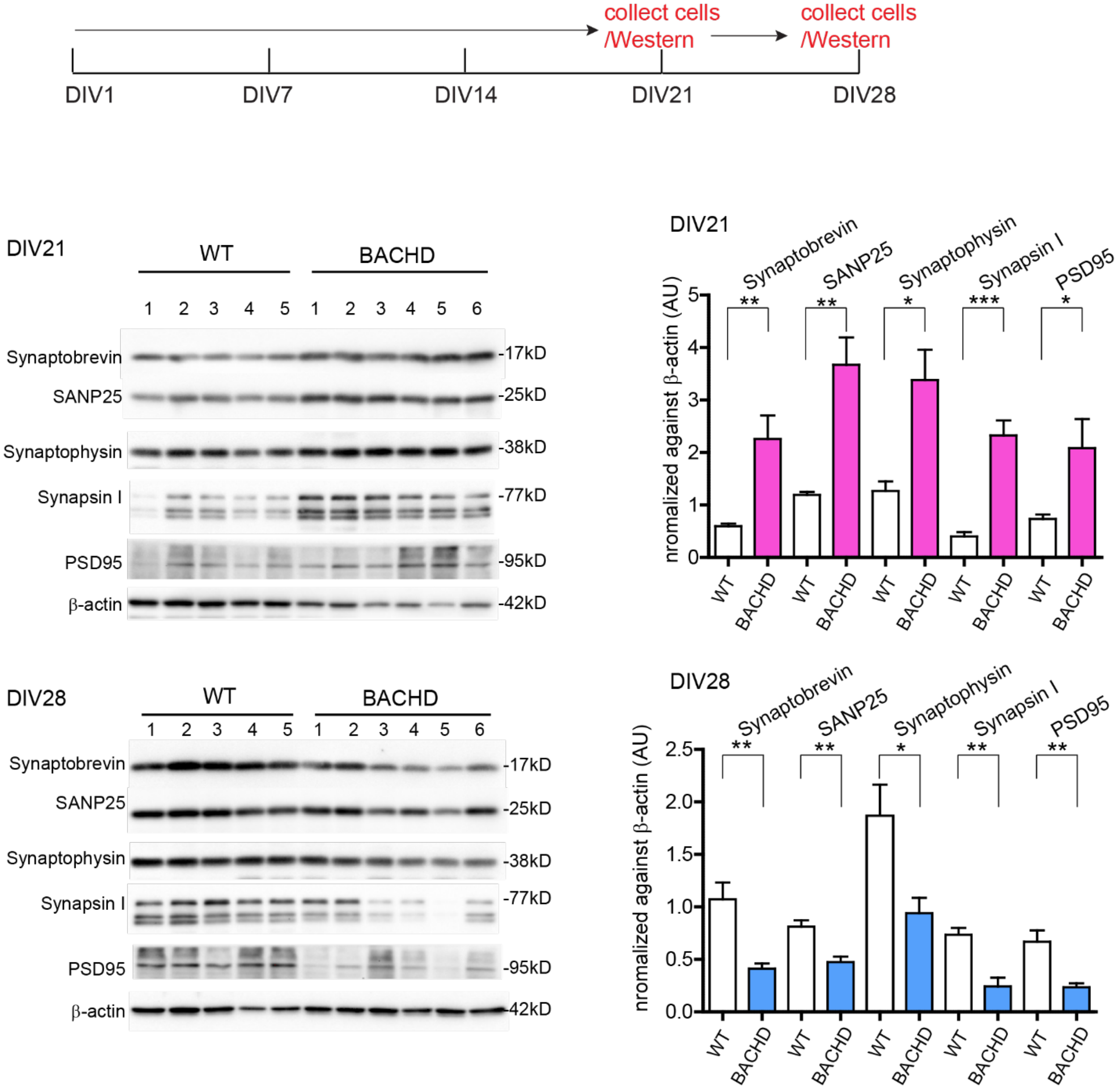

collect cells /Western
collect cells /Western
DIV1
DIV7
DIV14
DIV21
DIV28
DIV21
WT
BACHD
1 2 3 4 5 1 2 3 4 5 6
Synaptobrevin
SANP25
Synaptophysin
Synapsin I
PSD95
β-actin
-17kD
-25kD
-38kD
-77kD
-95kD
-42kD
DIV21
Synaptobrevin
SANP25
Synaptophysin
Synapsin I
PSD95
** ** * *** *
nromalized against β-actin (AU)
0 1 2 3 4 5
WT BACHD WT BACHD WT BACHD WT BACHD WT BACHD
DIV28
WT
BACHD
1 2 3 4 5 1 2 3 4 5 6
Synaptobrevin
SANP25
Synaptophysin
Synapsin I
PSD95
β-actin
-17kD
-25kD
-38kD
-77kD
-95kD
-42kD
DIV28
Synaptobrevin
SANP25
Synaptophysin
Synapsin I
PSD95
** ** * ** **
nromalized against β-actin (AU)
0.0 0.5 1.0 1.5 2.0 2.5
WT BACHD WT BACHD WT BACHD WT BACHD WT BACHD

Fig S2

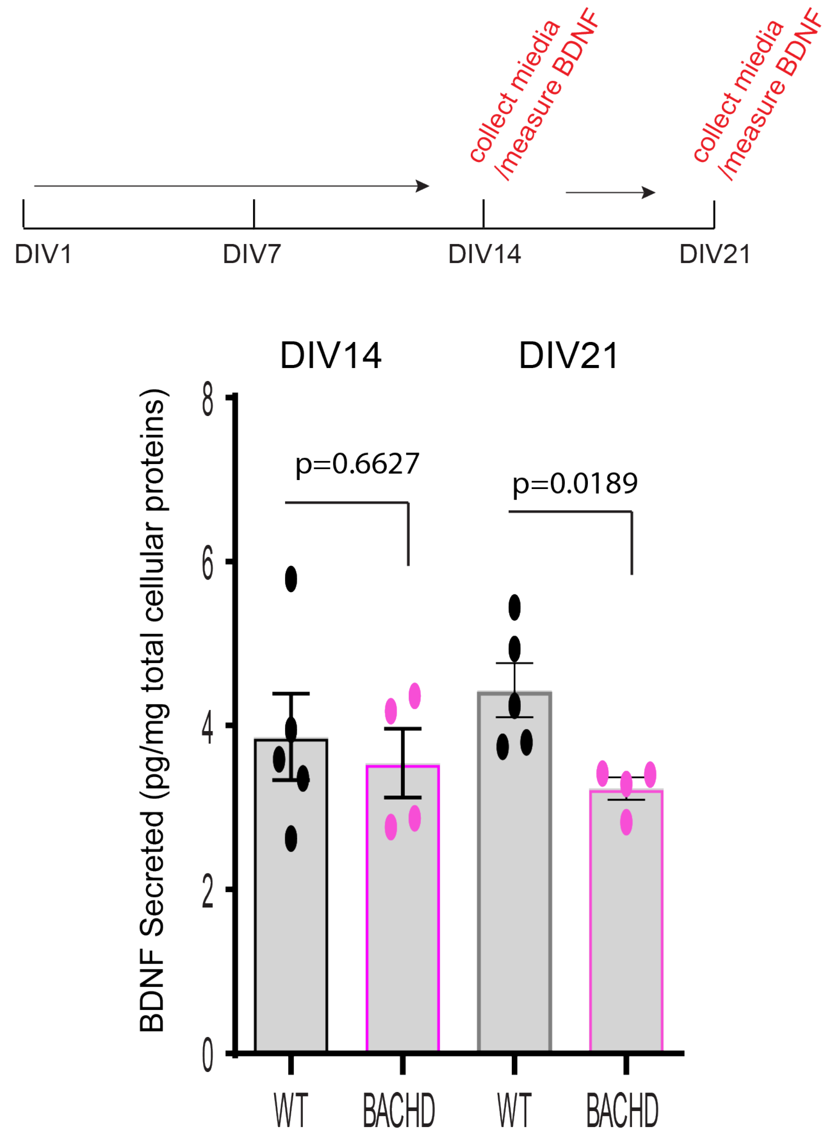

collect miedia
/measure BDNF
collect miedia
/measure BDNF
DIV1
DIV7
DIV14
DIV21
DIV14
DIV21
BDNF Secreted (pg/mg total cellular proteins)
8
6
4
2
0
p=0.6627
p=0.0189
WT
BACHD
WT
BACHD

Fig S3

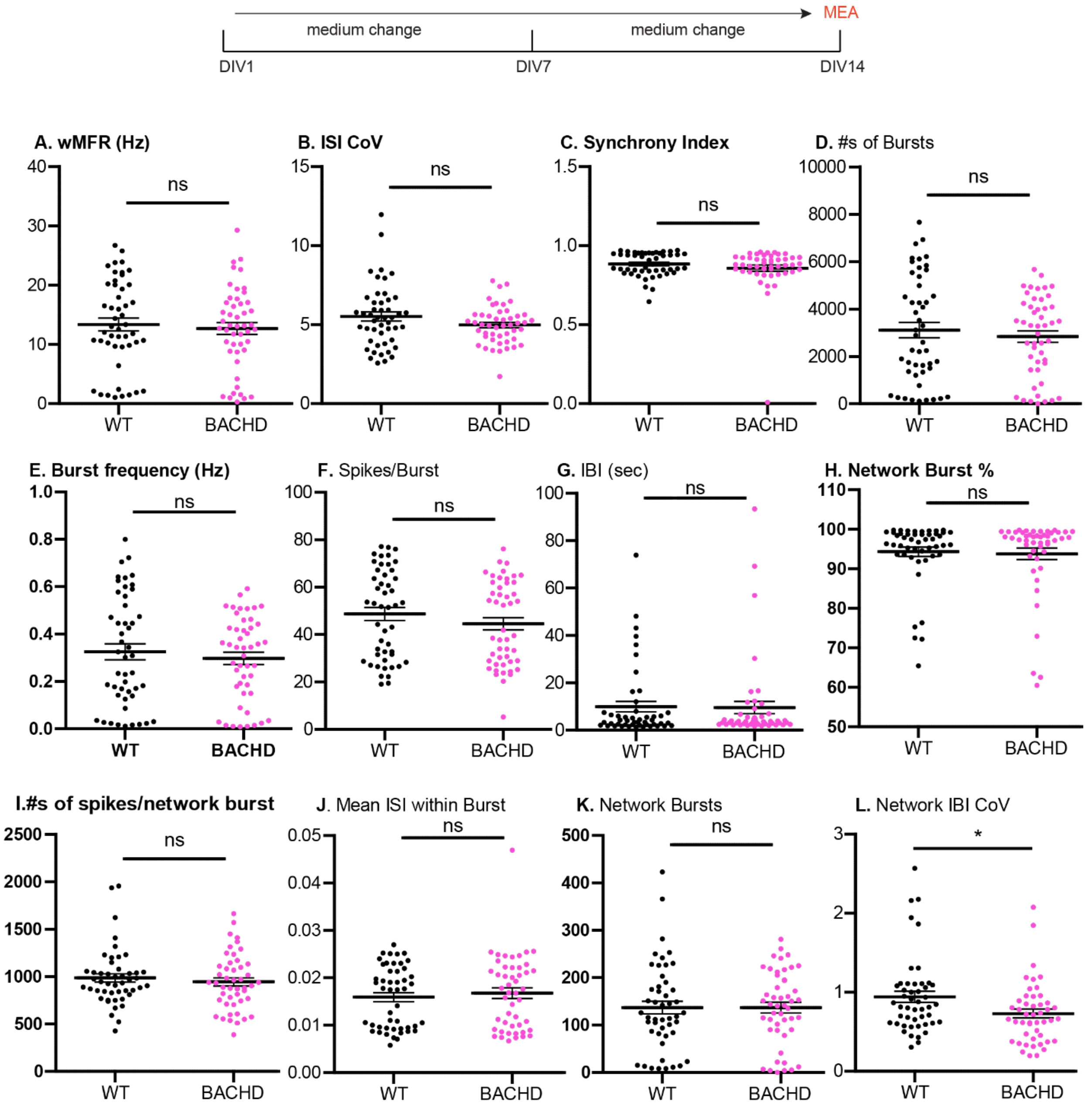

MEA
medium change
medium change
DIV1
DIV7
DIV14
A. wMFR (Hz)
B. ISI CoV
C. Synchrony Index
D. #s of Bursts
E. Burst frequency (Hz)
F. Spikes/Burst
G. IBI (sec)
H. Network Burst %
I.#s of spikes/network burst
J. Mean ISI within Burst
K. Network Bursts
L. Network IBI CoV
ns
*
WT
BACHD

Fig S4

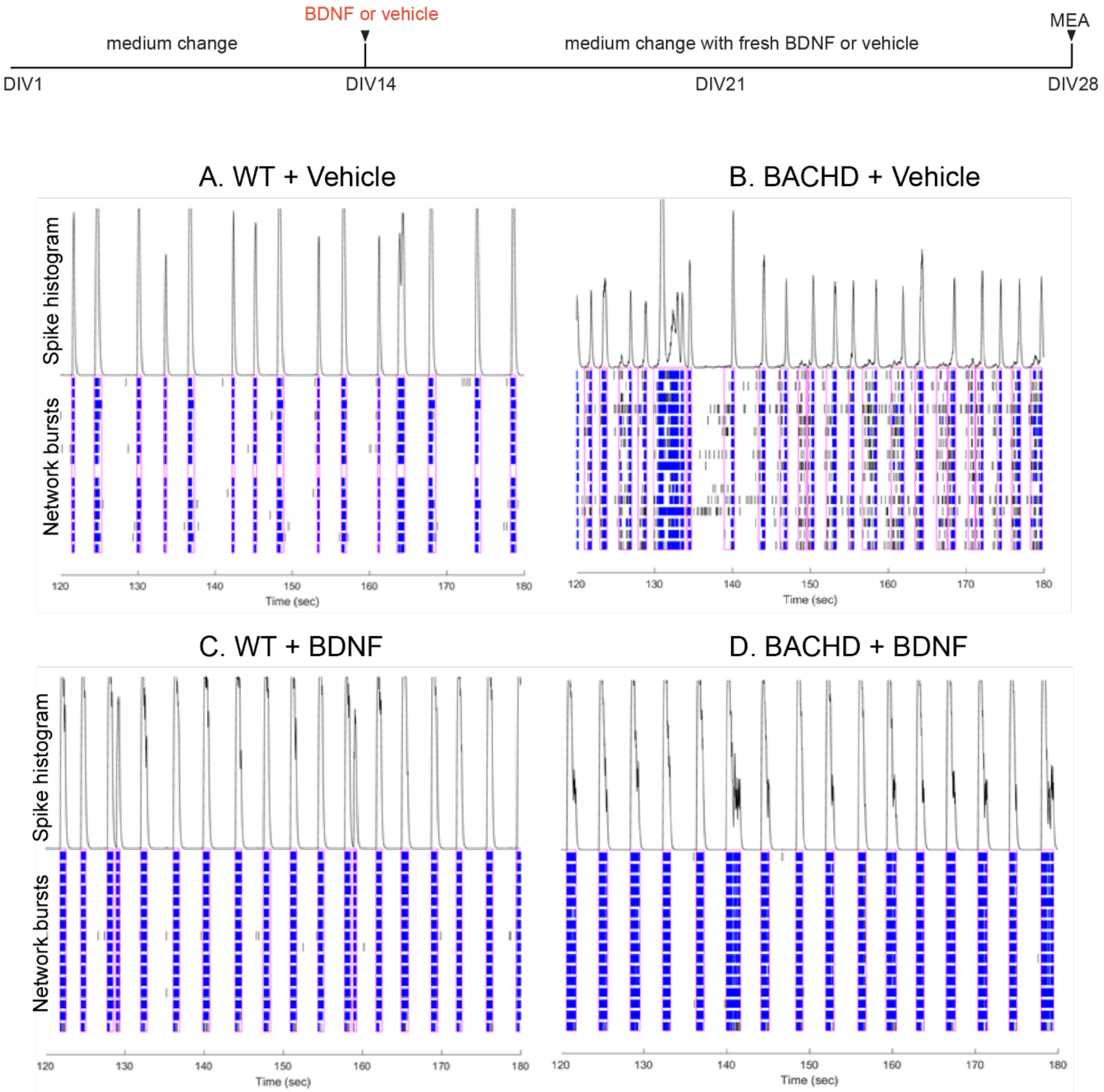
BDNF or vehicle
medium change
MEA
medium change with fresh BDNF or vehicle
DIV1
DIV14
DIV21
DIV28
A. WT + Vehicle
B. BACHD + Vehicle
C. WT + BDNF
D. BACHD + BDNF
Spike histogram
Network bursts
Time (sec)
120
130
140
150
160
170
180

Fig S5

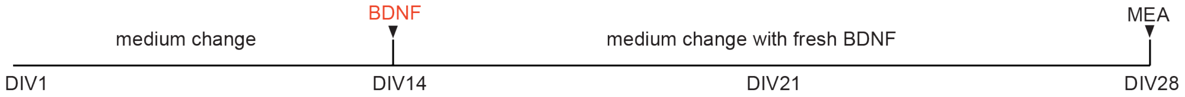
BDNF
MEA
medium change
medium change with fresh BDNF
DIV1
DIV14
DIV21
DIV28

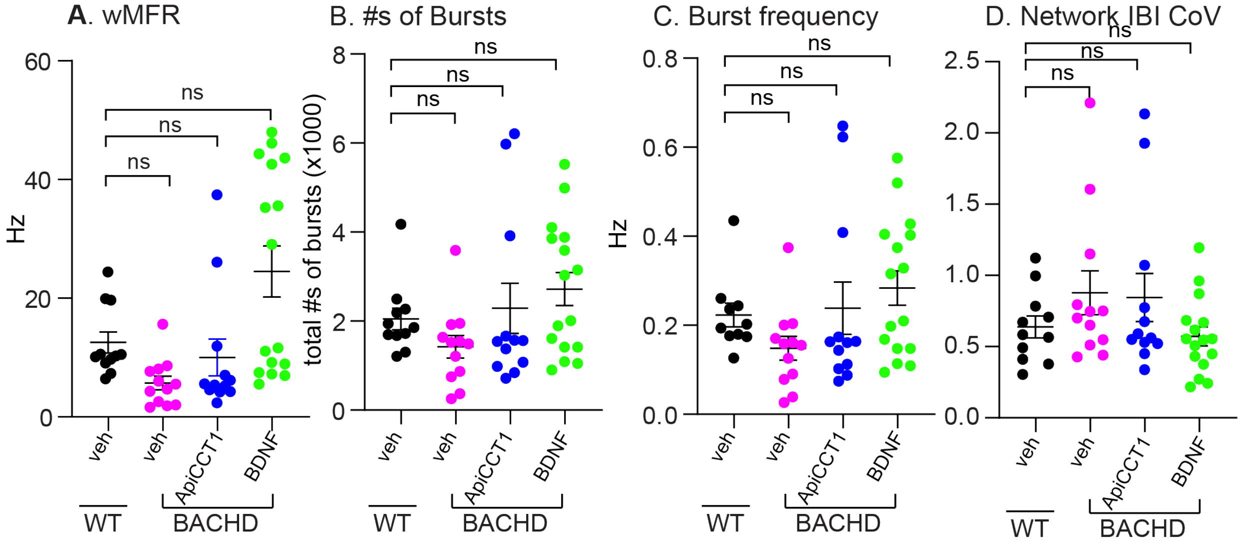
A. wMFR
B. #s of Bursts
C. Burst frequency
D. Network IBI CoV
Hz
total #s of bursts (x1000)
ns
veh
veh
ApiCCT1
BDNF
WT
BACHD

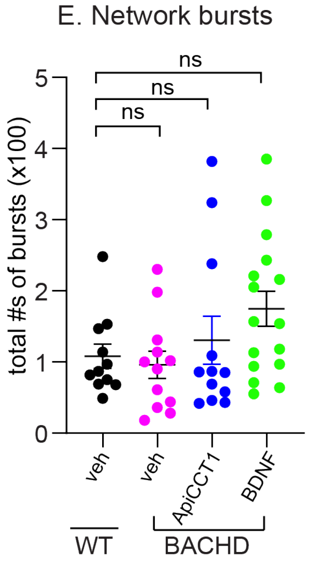
E. Network bursts
total #s of bursts (x100)
ns
veh
veh
ApiCCT1
BDNF
WT
BACHD
